# Cardiomyocyte-derived USP20 mitigates myocardial ischemia/reperfusion injury through deubiquitinating GRP78

**DOI:** 10.7150/thno.132067

**Published:** 2026-06-17

**Authors:** Zhenfeng Cheng, Lingfeng Zhong, Miaomiao Ying, Ziyi Huang, Zexin Yang, Wenli Zhang, Zhouqing Huang, Naijin Zhang, Xudong Chen, Xiaoxi Fan, Yucong Zhang, Ruihan Zheng, Keke Ye, Shuang Lin, Weijian Huang, Guangju Zhao, Shanshan Dai

**Affiliations:** 1 Huzhou Central Hospital, Affiliated Central Hospital of Huzhou University, Huzhou, Zhejiang, 313000, China.; 2 The Key Laboratory of Cardiovascular Disease of Wenzhou, Department of Cardiology, The First Affiliated Hospital of Wenzhou Medical University, Wenzhou, Zhejiang, 325000, China.; 3 Department of Pathology, First Affiliated Hospital of Wenzhou Medical University, Wenzhou, Zhejiang, 325000, China.; 4 Department of Emergency, The First Affiliated Hospital of Wenzhou Medical University, The Key Laboratory of Emergency and Disaster Medicine of Wenzhou, Wenzhou, Zhejiang, 325000, China.; 5 Department of Cardiovascular Medicine, Ruijin Hospital, Shanghai Jiao Tong University School of Medicine, 200000, Shanghai, China.; 6 Department of Cardiology, First Hospital of China Medical University, Shenyang City, 110000, China.; 7 Department of Cardiology, Ningbo Hangzhou Bay Hospital, Ningbo, Zhejiang, 315000, China.; 8 Department of Cardiology, Ningbo Medical Center Lihuili Hospital, Ningbo, Zhejiang, 315000, China.

**Keywords:** deubiquitinating enzyme, ubiquitin-specific peptidase 20, myocardial ischemia/reperfusion injury, cardiomyocyte, glucose-regulated protein 78

## Abstract

Myocardial ischemia/reperfusion (I/R) injury remains a major clinical challenge characterized by cardiomyocyte loss and adverse remodeling after reperfusion. Ubiquitin-Specific Peptidase 20 (USP20), a deubiquitinase involved in cellular stress responses, has not been fully characterized in the ischemic heart. This study aimed to investigate the function and mechanism of cardiomyocyte-derived USP20 in myocardial I/R injury.

**Methods:**

Myocardial I/R models and hypoxia/reoxygenation (H/R)-treated cardiomyocytes were used to evaluate USP20 expression and function. Single-cell transcriptomic analysis, cardiomyocyte-specific USP20 knockout, and USP20 overexpression models were applied. Co-immunoprecipitation, LC-MS/MS, ubiquitination assays, site-directed mutagenesis, and rescue experiments were performed to define the underlying mechanism.

**Results:**

USP20 expression was markedly decreased in I/R-injured mouse hearts and H/R-treated cardiomyocytes, and USP20 was predominantly localized in cardiomyocytes. Cardiomyocyte-specific USP20 deletion aggravated myocardial injury, adverse cardiac remodeling, and cardiac dysfunction after I/R, whereas USP20 overexpression conferred significant cardioprotection. Mechanistically, USP20 directly interacted with glucose-regulated protein 78 (GRP78) and removed K63-linked polyubiquitin chains from GRP78 at K602 through its C154 active site. This deubiquitination activated GRP78, promoted adaptive endoplasmic reticulum stress responses, and reduced myocardial injury.

**Conclusions:**

These findings identify a previously unrecognized USP20-GRP78 regulatory axis that regulates endoplasmic reticulum stress responses and limits myocardial damage during I/R. USP20 may represent a potential therapeutic target for myocardial I/R injury.

## Introduction

Myocardial ischemia-reperfusion (I/R) injury is characterized by aggravated cellular damage triggered by the reinstatement of blood circulation to previously ischemic heart tissue, poses a critical challenge in the clinical management of acute coronary syndromes such as myocardial infarction 1. While this restoration is essential to prevent irreversible damage to heart tissue, it paradoxically induces further injury through a complex cascade of biochemical and cellular events. This intricate cascade of events, initiated by the initial oxygen deprivation during ischemia, is marked by oxidative stress, calcium dyshomeostasis, mitochondrial impairment, ER stress, and cellular injury, ultimately leading to impaired cardiac performance and irreversible myocardial injury 1. Therefore, defining endogenous protective mechanisms in I/R injury is essential for developing improved cardioprotective strategies.

Cardiomyocytes are terminally differentiated cells with minimal regenerative potential; thus, they rely heavily on protein quality-control systems to preserve cellular function under stress. The stability and function of proteins are precisely regulated by post-translational modifications. The ubiquitin-proteasome system (UPS) represents a critical form of post-transcriptional post-transcriptional regulation in eukaryotic cells 2. The UPS comprises two reversible processes, ubiquitylation and deubiquitylation. Ubiquitination is a process in which ubiquitin, a small regulatory protein, is covalently conjugated to target substrate proteins 3. This modification can signal for protein degradation, modify protein activity, or affect protein-protein interactions 4. Deubiquitylation serves as the reverse process, where specific enzymes known as deubiquitinating enzymes (DUBs) catalyze the hydrolytic cleavage of ubiquitin conjugates from substrate proteins 5. This mechanism is crucial for regulating the UPS and maintaining protein homeostasis 6. To date, researchers have identified over 100 DUBs categorized in seven different families, with the USP family being the largest among them 7.

Ubiquitin-specific peptidase 20 (USP20), a deubiquitylating enzyme belonging to the USP family, serves as a critical regulator of protein ubiquitination dynamics, and regulates a spectrum of cellular processes, including cell survival, proliferation, and inflammation 8. USP20 is recognized as a significant regulator of multiple diseases with profound implications for cancer 9-11, inflammatory disorders 12, metabolic dysfunctions 13, and neurological diseases 14. It has been reported that USP20 can mitigate ischemic stroke in murine models by suppressing neuroinflammation and neuronal death via modulation of the PTEN signaling pathway 15. In addition, USP20 attenuates atherosclerosis by deubiquitinating RIPK1 to suppress tumor necrosis factor-triggered smooth muscle cell inflammation 16. However, the involvement of USP20 in myocardial I/R injury remains unexplored. Our preliminary observations showed a clear reduction of USP20 after myocardial I/R injury, suggesting a potential role in this pathological process.

In light of these findings, we investigated the function of USP20 in myocardial I/R injury and explored the molecular mechanisms involved. This research may provide fundamental insights into utilizing USP20 as a viable target for intervention in such pathological conditions.

## Materials and Methods

### Animal experiments

All animal protocols were reviewed and approved by the Laboratory Animal Ethics Committee and Laboratory Animal Centre of the First Affiliated Hospital of Wenzhou Medical University (NO. WYYY-AEC-YS-2023-0498). Cardiac-specific USP20 knockout (USP20^fl/fl^ Myh6-Cre, USP20-CKO) male mice, aged 8-week-old, with a C57BL/6J genetic background, along with genetically matched wild-type littermates (USP20^fl/fl^) were obtained from Gem Pharmatech Co., Ltd. Mice were maintained under specific pathogen-free conditions at 25 °C with free access to food and water. Group allocation was randomized, and investigators were blinded during experimental assessment. All procedures followed Directive 2010/63/EU of the European Parliament.

Myocardial I/R injury was generated by temporary ligation of the left anterior descending (LAD) coronary artery followed by reperfusion, as described previously 17. Briefly, the LAD was exposed and identified, after which a 7-0 silk suture was placed around the artery approximately 2 mm distal to its origin and tightened to induce occlusion. Myocardial ischemia was confirmed by visible blanching of the cardiac tissue downstream of the ligation site. After 30 min of ischemia, reperfusion was achieved by gently releasing the ligature. Acute injury was assessed after 24 h of reperfusion, whereas chronic I/R injury was evaluated after 4 weeks. To control for surgical effects, sham-operated animals underwent an identical procedure as the model group, excluding the LAD occlusion. At the endpoint, mice were euthanized with an overdose of sodium pentobarbital (Euthanyl, 100 mg/kg, intraperitoneally), and serum as well as infarct and non-infarct myocardial tissues were collected.

To induce cardiomyocyte-specific USP20 overexpression in mouse hearts, an adeno-associated virus serotype 9 (AAV9) vector driven by the cardiac troponin T promoter (cTnT) was constructed to express USP20 (AAV9-cTnT-USP20^oe^) or the corresponding empty vector control (AAV9-cTnT-EV) (Genechem Co., Ltd., Shanghai, China). Mice were administered a single tail-vein injection containing 1×10^11^ viral genomes four weeks prior to myocardial I/R surgery.

### Echocardiography

Echocardiography was performed under 3% isoflurane anesthesia with mice placed on a temperature-controlled platform. M-mode images were obtained from the standard short-axis view, and ejection fraction (EF) and fractional shortening (FS) were measured using a Vevo 3100 small-animal ultrasound system (FUJIFILM VisualSonics, Canada).

### Assessment of area at risk (AAR) and infarcted size (INF)

Measurement of the AAR and INF is critical for assessing myocardial injury and treatment efficacy. The AAR represents ischemic myocardium, while INF indicates the extent of irreversible myocardial damage. Following the induction of myocardial I/R injury, the LAD artery was re-ligated. Subsequently, a volume of 1 ml of a 2% Evans Blue solution (E2129, Sigma-Aldrich, St. Louis, MO) was administered into the inferior vena cava via an abdominal incision. Once coloration change was observed, the heart was promptly excised and placed in pre-cooled PBS to remove residual blood from its tissue. Following Evans blue staining, hearts were rapidly harvested, washed with ice-cold PBS, blotted dry, and frozen at -20 °C for 15 min. Cardiac tissues were then sectioned transversely into 1-mm slices. Non-ischemic regions were stained blue, whereas the AAR remained unstained. The slices were subsequently incubated in 1% TTC solution (Solarbio, Beijing, China) at 37 °C for 15 min in the absence of light and fixed in 4% paraformaldehyde for 2 h. Infarcted myocardium was identified as the pale white region within the AAR.

### Serum biochemical analysis

Serum was collected after centrifugation of blood samples at 3000 g for 15 min. Commercial kits were used to determine serum levels of cardiac troponin T (cTnT; Elabscience, Wuhan, China), lactate dehydrogenase (LDH; Solarbio, Beijing, China), creatine kinase-MB (CK-MB; Jiancheng Bioengineering Institute, Nanjing, China), and atrial natriuretic peptide (ANP; Xitang, Shanghai, China) according to the manufacturers’ protocols.

### Histopathological staining

Paraffin-embedded cardiac sections were stained using a Masson staining kit (G1340, Solarbio, Beijing, China). The TUNEL staining kit (C1086, Beyotime, Shanghai, China) was employed for the staining of frozen heart samples. Ultimately, the specimens were examined under a microscope.

### Cell experiments

Neonatal rat primary cardiomyocytes (NRPCs) were prepared from ventricular tissues of newborn Sprague-Dawley rats, whereas adult ventricular cardiomyocytes (AVCMs) were isolated from adult murine hearts. NIH/3T3 and HL-1 cell lines were purchased from the Shanghai Institute of Biochemistry and Cell Biology (Shanghai, China). All cells were cultured in DMEM supplemented with 10% fetal bovine serum and 1% penicillin/streptomycin under standard conditions. To establish the H/R model, NRPCs were incubated in glucose-free DMEM under hypoxic conditions (1% O₂, 5% CO₂, and 94% N₂) for 4 h, followed by reoxygenation in high-glucose DMEM containing 10% FBS under normoxic conditions (21% O₂, 5% CO₂, and 74% N₂) for an additional 6 h. Control cells were cultured under normoxia for the same duration.

To evaluate the involvement of USP20 in H/R-induced cardiomyocyte injury, gain- and loss-of-function approaches were performed. USP20 overexpression was achieved by transfecting cells with a USP20 expression plasmid using Lipofectamine 3000, while USP20 knockdown was induced using specific siRNA diluted in Opti-MEM medium. Cells transfected with empty vector or control siRNA served as corresponding controls. Functional experiments were conducted 24 h after transfection.

### Cell viability assessment

Cell viability was assessed using the cell counting kit (CCK-8, C0038, Beyotime, Shanghai, China) and the LDH cytotoxicity assay kit (C0016, Beyotime, Shanghai, China), respectively, following the manufacturer's instructions.

### Superoxide dismutase (SOD) activity and malondialdehyde (MDA) concentration detection

After lysis and centrifugation of cardiomyocytes, the supernatant was collected for biochemical assays. MDA content and SOD activity were determined using assay kits (S0131S and S0101S, Beyotime, Shanghai, China) according to the supplied protocols.

### Dihydroethidium (DHE) detection

Intracellular DHE fluorescence was measured with a DHE assay kit (50102ES02, Yeasen Biotechnology). Cardiomyocytes were incubated with the DHE solution for 30 min at 37 °C. After three washes, fluorescence detection was performed using a fluorescence microscope.

### Human samples

Human myocardial tissues were obtained according to our previously published protocol 17. All procedures involving human samples were reviewed and approved by the Ethics Committee of the First Affiliated Hospital of Wenzhou Medical University (Approval No. KY2022-156) and conducted in accordance with the principles outlined in the Declaration of Helsinki. Written informed consent was obtained from the patients’ immediate family members.

### Real-time quantitative polymerase chain reaction (RT-qPCR)

Total RNA was isolated with TRIzol reagent, followed by reverse transcription using HiScript III All-in-one RT SuperMix (Vazyme, China). Quantitative PCR analysis was carried out using ChamQ Universal SYBR qPCR Master Mix on a real-time PCR platform according to the manufacturer’s instructions. Amplification was performed under the following conditions: initial denaturation at 95 °C for 10 min, followed by 40 amplification cycles consisting of 95 °C for 15 s and 60 °C for 1 min. Gene expression levels were normalized to *β-actin* and analyzed using the 2^-ΔΔCt^ method. Primer sequences designed with Primer-BLAST are provided in Table S1.

### Western blot

Total proteins were extracted from cultured cardiomyocytes and myocardial tissues, and protein concentrations were quantified using a BCA protein assay kit. Equivalent amounts of protein samples were resolved by SDS-PAGE and transferred to PVDF membranes. After blocking with 5% skim milk at room temperature for 1 h, the membranes were incubated overnight at 4 °C with the indicated primary antibodies, including USP20 (ab72225, 1:1000), TGF-β (21898-1-AP, 1:1000), Col-1 (14695-1-AP, 1:1000), p-PERK (3179S, 1:1000), IRE1α (3294S, 1:1000), ATF6 (65880S, 1:1000), p-MLKL (37333S, 1:1000), p-RIPK1 (53286S, 1:1000), p-RIPK3 (91702S, 1:1000), GRP78 (66574-1-Ig, 1:1000), β-actin (AC026, 1:10000) and GAPDH (MB001,1:10000). Membranes were then incubated with corresponding secondary antibodies for 1 h at room temperature. Protein bands were detected using an enhanced chemiluminescence reagent following TBST washes.

### Co-immunoprecipitation (Co-IP) assays

For co-immunoprecipitation experiments, proteins were prepared from myocardial tissues or primary cardiomyocytes using RIPA lysis buffer. Cell or tissue lysates were incubated with the indicated antibodies at 4 °C overnight, while a portion of the lysate was retained as the input control. Antibody-protein complexes were subsequently precipitated using protein G-Sepharose beads, washed with PBS, and subjected to immunoblot analysis.

### RNA sequencing

For single-cell RNA sequencing analysis, heart tissues from sham and I/R mice were enzymatically digested to generate single-cell suspensions. Cells isolated from 3-4 hearts within each group were combined and processed using the 10X Genomics Chromium platform for single-cell capture and library construction with the Chromium Single Cell 3’ kit. Subsequent cDNA amplification, library generation, and sequencing were completed by LC-BIO Technologies (Hangzhou, China).

For bulk RNA sequencing, total RNA was isolated from myocardial tissues using TRIzol reagent. RNA libraries were prepared and sequenced on the Illumina NovaSeq 6000 platform by LC-Bio Technology Co., Ltd. (Hangzhou, China) according to standard protocols.

### Proteomic analysis by LC-MS/MS

NRPCs were transfected with Flag-tagged USP20 plasmids prior to H/R treatment. Protein complexes associated with USP20 were immunoprecipitated using anti-Flag antibodies coupled to protein G-Sepharose beads. The precipitated proteins were subsequently released with SDT lysis buffer, digested into peptides via the filter-aided sample preparation (FASP) protocol, and analyzed by liquid chromatography–tandem mass spectrometry (LC-MS/MS) at PTM Bio Co., Ltd. (Zhejiang, China).

### Statistical analysis

Results are presented as mean ± SD. Statistical analyses were conducted using GraphPad Prism 8.0 software. The sample number (n) refers to independent biological replicates. For cell-based experiments, data were considered to follow a normal distribution because measurements were obtained from large populations of cultured cells. Animal experiments included at least six mice in each group. Distribution normality was evaluated using the Shapiro-Wilk test. Comparisons between two groups were analyzed using unpaired Student’s t-tests, while differences among multiple groups were assessed by one-way ANOVA followed by Tukey’s multiple-comparison test. Statistical significance was defined as P < 0.05.

## Results

### Identification of cardiomyocyte-derived USP20 as a critical factor in myocardial I/R injury

**Figure 1:**
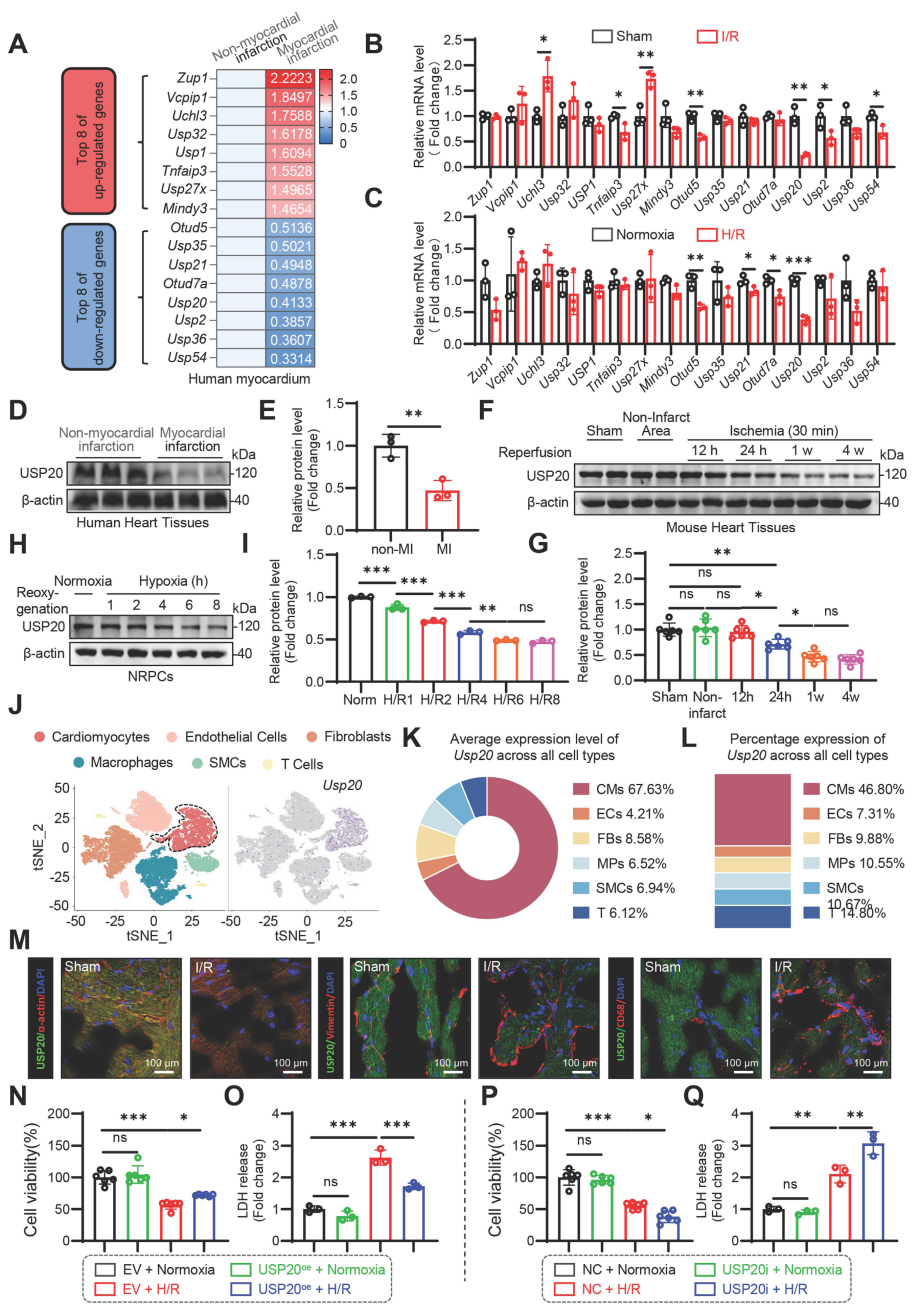
** Identification of Cardiomyocyte-Derived USP20 as a Critical Factor in Myocardial Ischemia/Reperfusion (I/R) Injury.** (**A**) Human heart tissue samples were collected from a patient with myocardial infarction as well as from a control sample for RNA sequencing. The heat map illustrating the top 8 up-regulation or down-regulation mRNA expression levels of UPS family members in both non-infarcted and infarcted regions. (**B, C**) Myocardial tissue in mice was subjected to 30 min of ischemia, followed by a subsequent period of 24 h of reperfusion, in order to induce I/R injury. The neonatal rat primary cardiomyocytes (NRPCs) were cultivated in glucose- and FBS deprived DMEM under hypoxic conditions for a duration of 4 h to induce hypoxic injury. Subsequently, the medium was replaced with a high-glucose medium containing 10% FBS, after which the cardiomyocytes were incubated under normoxic condition for an additional 6 h to induce hypoxia/reoxygenation (H/R) injury. The mRNA levels of deubiquitinating enzymes belonging to USP family, which exhibited differentially expression in RNA transcriptome sequencing as illustrated in Figure 1A, were analyzed in mouse heart tissue subjected to I/R injury (**B**) and in NRPCs induced by H/R injury (**C**). n = 3. (**D, E**) The protein expression levels of USP20 in both patients with myocardial infarction as well as control samples assessed by western blot analysis (**D**) and the statistical results (**E**). n = 3. (**F, G**) The protein expression levels of USP20 in mouse heart tissue from the sham group, as well as the I/R group in both non-infarcted and myocardial injury regions at various reperfusion time points assessed by western blot (**F**) and the statistical results (**G**). n = 6. (**H, I**) The protein expression levels of USP20 in NRPCs subjected to normoxia and hypoxia treatments followed by different reoxygenation time points evaluated by western blot (**H**) and the statistical results (**I**). n = 3. (**J**) Single-cell RNA sequencing (scRNA-seq) was conducted on heart tissues obtained from mouse subject to sham or I/R treatments. For each group, single-cell suspensions from 3 hearts were pooled as 1 sample. Left, the tSNE dimensional reduction showing 6 main cell types of heart, including cardiomyocytes (CM), endothelial cells (EC), fibroblasts (FB), macrophages (MP), smooth muscle cells (SMCs) and T cells. Approximate 10224 single heart cells in sham group and 7018 single heart cells in I/R group were analyzed. Right, biaxial scatter plot showing the expression pattern of *Usp20* in sham and I/R group. (**K**) Quantification of the average expression level of Usp20 across different cardiac cell types based on scRNA-seq analysis. Quantification of the percentage of Usp20-expressing cells among different cardiac cell populations based on scRNA-seq analysis. (**M**) Representative images of USP20 immunofluorescence staining of mouse myocardial tissue from Sham and I/R group. (**N, O**) Assessment of cell viability (**N**) and lactate dehydrogenase (LDH) release (**O**) in NRPCs overexpressing USP20 subjected to normoxia or H/R. n = 6. (**P, Q**) Assessment of cell viability (**P**) and LDH release (**Q**) in NRPCs down-regulated of USP20 subjected to normoxia or H/R. n = 6. Data are expressed as the mean ± standard deviation (SD). ***, P < 0.001; **, P < 0.01; *, P < 0.05; ns, P > 0.05, ns: no differences. Student’s t-test for B, C and E; One-way ANOVA followed by Tukey's post hoc test for G, I, N, O, P and Q.

Transcriptomic sequencing was performed to examine the expression landscape of deubiquitinating enzymes (DUBs) in myocardial infarction and non-failing human heart tissues. Differential expression analysis identified the top eight upregulated and top eight downregulated DUB family members in infarcted hearts (**Figure 1A**). These candidate genes were subsequently examined by qPCR in myocardial tissues subjected to I/R as well as in cardiomyocytes exposed to H/R. Among them, USP20 showed a pronounced reduction at the mRNA level in both experimental settings (**Figure 1B-C**). Consistently, analysis of human infarct samples demonstrated markedly lower USP20 protein and transcript levels compared with control myocardial tissues (**Figure 1D-E and Figure S1A**). Additionally, we assessed the temporal expression profile of USP20 following reperfusion of myocardial tissue in mice. Our findings indicated a time-dependent decrease in both USP20 protein and mRNA levels within I/R-induced myocardial tissue (**Figure 1F-G** and **Figure S1B**) as well as in H/R-induced cardiomyocytes (**Figure 1H-I** and **Figure S1C**). However, this decrease in USP20 was not detected in fibroblasts (**Figure S1D-F**). To avoid false-negative results arising from cardiomyocyte death, adult cardiomyocytes were isolated and USP20 expression levels were assessed under conditions of consistent cardiomyocyte cell numbers. Consistent with these findings, USP20 expression was markedly decreased after myocardial I/R injury (**Figure S1G**). Single-cell RNA sequencing further revealed six major cardiac cell populations in sham and I/R hearts, including cardiomyocytes, endothelial cells, fibroblasts, macrophages, smooth muscle cells, and T cells. Among these populations, USP20 expression was predominantly detected in cardiomyocytes. Moreover, I/R injury was accompanied by a reduction in both cardiomyocyte abundance and USP20 expression levels (**Figure 1J-L**). Immunofluorescence staining further corroborated that the reduction in USP20 was predominantly observed within cardiomyocytes, whereas its expression levels in fibroblasts and macrophages showed no notable alterations (**Figure 1M**). Accordingly, we further investigated the functional role of USP20 in cardiomyocytes under H/R conditions. USP20 overexpression markedly attenuated H/R-induced cardiomyocyte injury (**Figure 1N-O**), whereas USP20 silencing further aggravated cellular damage following H/R stimulation (**Figure 1P-Q**). Through the above research, we have preliminarily established the role of cardiomyocyte-derived USP20 in myocardial I/R injury.

### Cardiomyocyte-specific USP20 deletion aggravates myocardial I/R-induced cardiac injury and remodeling

**Figure 2:**
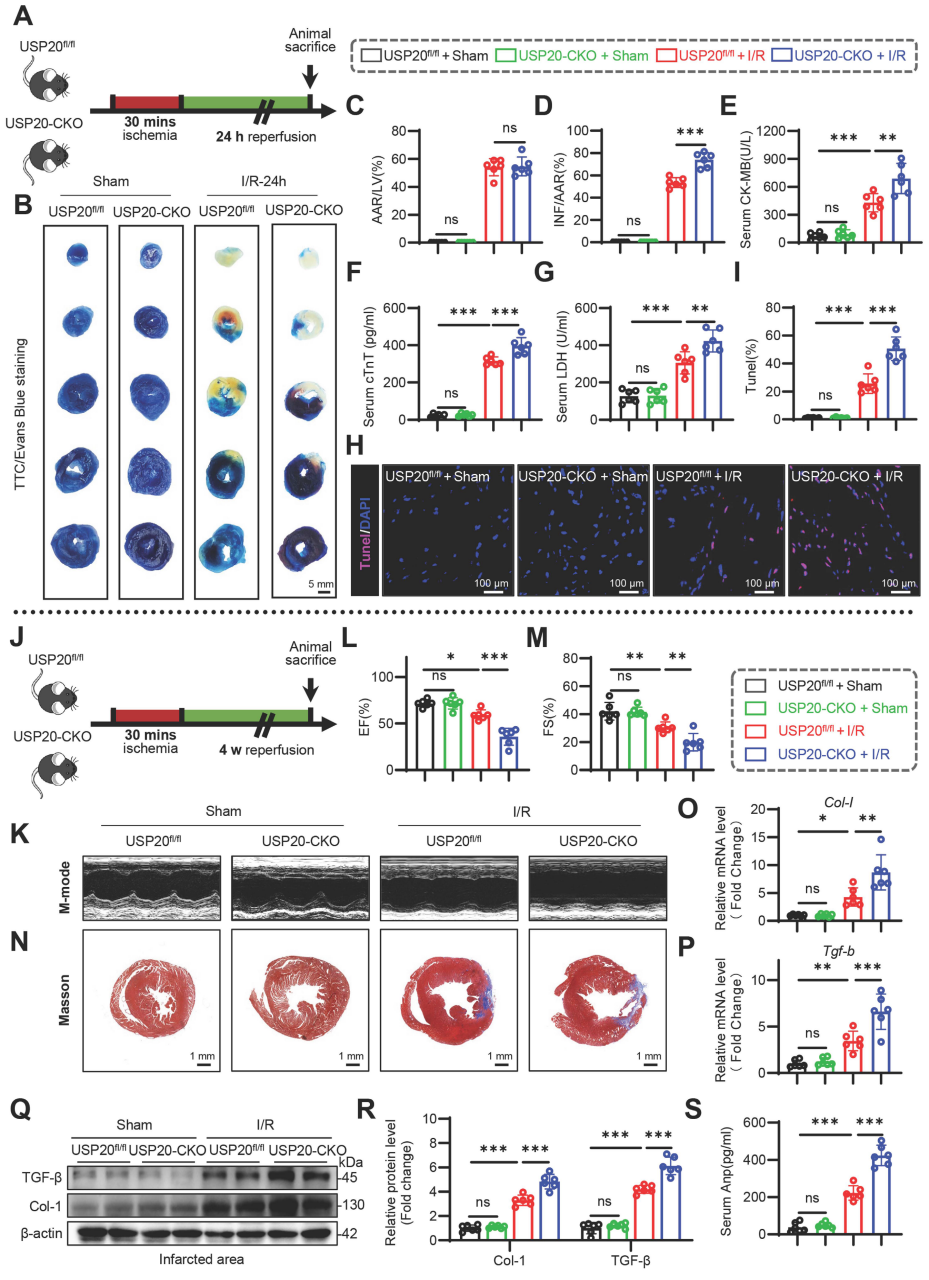
** Cardiomyocyte-specific USP20 Deletion Aggravates Myocardial I/R-induced Cardiac Injury and Remodeling.** (**A**) Schematic diagram of acute myocardial I/R injury mouse model. (**B-D**) Representative images of triphenyl tetrazolium chloride (TTC)/Evans Blue staining of heart tissue (**B**) and the statistical results of the ratio of area at risk (AAR) to left ventricular area (**C**) and infarct size (INF) to AAR (**D**). Scale bar 50 mm. (**E-G**) Serum concentrations of creatine kinase isoenzyme MB (CK-MB) (**E**), cardiac troponin T** (**cTnT)(**F**), and LDH (**G**) in each group. (**H**, **I**) Representative images of TUNEL staining of myocardial tissue in each group (**H**) and the statistical results (**I**). (**J**) Schematic diagram of chronic myocardial I/R injury mouse model. (**K**) Representative M-mode echocardiography of mice in each group. Time stamp, 100 ms. Scale bar, 2mm. (**L, M**) Myocardial function parameters, ejection fraction (EF) (**L**) and fractional shortening (FS) (**M**) of mice measured by echocardiography. (**N**) Masson staining of myocardial tissue in each group. (**O, P**) The mRNA expression levels of *Col-I* (**O**) and *Tgf-β* (**P**) in mouse heart tissues of each group. (**Q, R**) The protein expression levels of COL-I and TGF-β in mouse heart tissue of each group (**Q**) and statistical results (**R**). (**S**) Serum level of atrial natriuretic peptide (ANP) in each group. Data are expressed as the mean ± SD. ***, P < 0.001; **, P < 0.01; *, P < 0.05; ns, P > 0.05, ns: no differences. n = 6. One-way ANOVA followed by Tukey's post hoc test for C, D, E, F, G, I, L, M, O, P, R and S.

To investigate the role of USP20 in myocardial ischemia/reperfusion injury, cardiomyocyte-specific USP20 knockout (USP20-CKO) mice were generated. Successful establishment of the knockout model was confirmed by genotyping analysis and evaluation of USP20 expression in multiple tissues as well as isolated cardiomyocytes (**Figure S2A-E**). USP20^fl/fl^ and USP20-CKO mice underwent either sham surgery or 30 min ischemia followed by 24 h reperfusion to establish acute I/R injury (**Figure 2A**). Cardiac-specific loss of USP20 significantly enlarged the infarct area after myocardial I/R, as demonstrated by Evans blue/TTC double staining (**Figure 2B-D**). Serum cardiac injury markers showed a similar pattern, with higher cTnT, CK-MB, and LDH levels in USP20-CKO mice after I/R (**Figure 2E-G**). Additionally, upon undergoing myocardial I/R injury, there was an increase in TUNEL-positive cells within the myocardium. This effect was significantly exacerbated in USP20-CKO mice that suffered from I/R injury (**Figure 2H-I**). Further investigation entailed establishing a chronic myocardial I/R injury model by subjecting animals to 30 min of coronary occlusion followed by four weeks of reperfusion (**Figure 2J**), thereby enabling assessment of USP20’s role in chronic cardiac I/R injury. The findings indicated that I/R injury resulted in cardiac dysfunction, whereas the deficiency of USP20 further exacerbated these functional impairments, as evidenced by the more pronounced reductions in EF and FS (**Figure 2K-M and Table S2**). Cardiac remodeling was assessed via Masson staining and detection of fibrosis-associated markers in heart tissue. Notably, Masson staining exhibited that USP20 deficiency significantly exacerbated myocardial fibrosis induced by I/R (**Figure 2N**). Correspondingly, elevated levels of fibrosis-associated markers including COL-1 and TGF-β were detected at both protein and mRNA levels in the heart tissue from I/R-induced USP20-CKO mice compared to those from their USP20^fl/fl^ counterparts undergoing similar treatment conditions (**Figure 2O-R**). Furthermore, serum ANP levels were markedly higher in the absence of USP20 during I/R events (**Figure 2S**). Taken together, USP20 deficiency markedly worsens myocardial injury and subsequent cardiac remodeling induced by I/R stress.

### USP20 modulates endoplasmic reticulum stress, necroptosis, and oxidative stress induced by H/R

**Figure 3:**
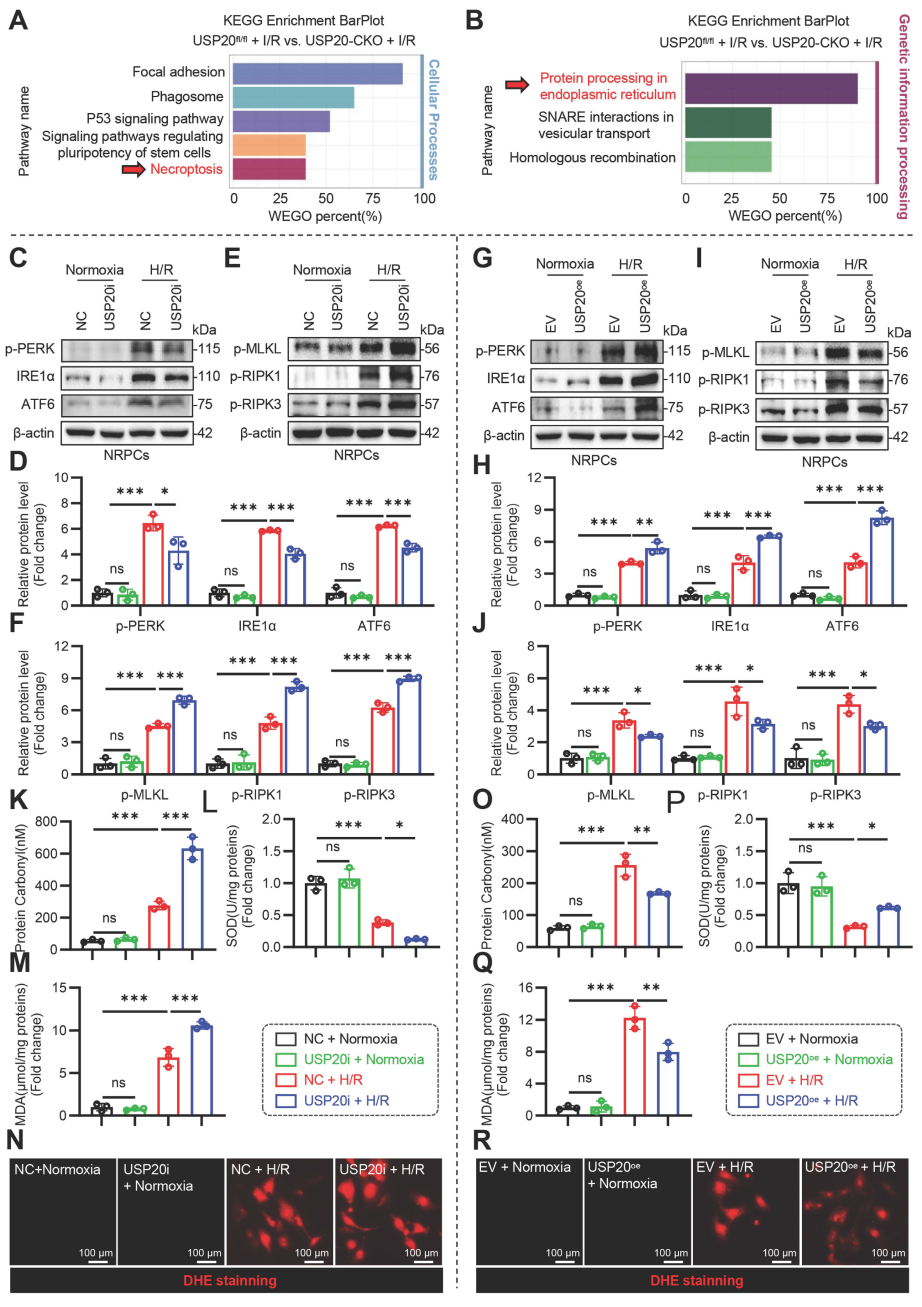
** USP20 Modulates Endoplasmic Reticulum Stress, Necroptosis, and Oxidative Stress Induced by Hypoxia/Reoxygenation (H/R).** (**A, B**) KEGG pathway analysis of the identified differentially expressed genes associated with cellular processes (**A**) and genetic information processing (**B**) in the RNA-seq results of mouse heart tissues from USP20^fl/fl^ + I/R group versus USP20-CKO + I/R group. (**C, D**) The protein expression levels of p-PERK, IRE1α, and ATF6 in NRPCs stimulated by H/R or normoxia following the silencing of USP20 expression utilizing siRNA (**C**) and the statistical results (**D**). n = 3. (**E, F**) The protein expression levels of p-MLKL, p-RIPK1 and p-RIPK3 in NRPCs stimulated by H/R or normoxia following the silencing of USP20 expression utilizing siRNA (**E**) and the statistical results (**F**). n = 3. (**G, H**) The protein expression levels of p-PERK, IRE1α, and ATF6 in NRPCs stimulated by H/R or normoxia following the overexpressing of USP20 utilizing plasmid (**G**) and the statistical results (**H**). n = 3. (**I, J**) The protein expression levels of p-MLKL, p-RIPK1 and p-RIPK3 in NRPCs stimulated by H/R or normoxia following the overexpressing of USP20 utilizing plasmid (**I**) and the statistical results (**J**). n = 3. (**K-N**) The levels of protein carbonylation (**K**), superoxide dismutase (SOD) activity (**L**), malondialdehyde (MDA) concentration (**M**) and dihydroethidium (DHE) (**N**) in NRPCs stimulated by H/R or normoxia following the silencing of USP20 expression utilizing siRNA. n = 3. (**O-R**) The levels of protein carbonylation (**O**), SOD activity (**P**), MDA concentration (**Q**) and DHE (**R**) in NRPCs stimulated by H/R or normoxia following the overexpressing of USP20 utilizing plasmid. n = 3. Data are expressed as the mean ± SD. ***, P < 0.001; **, P < 0.01; *, P < 0.05; ns, P > 0.05, ns: no differences. One-way ANOVA followed by Tukey's post hoc test for D, F, H, J, K, L, M, O, P and Q.

KEGG analysis suggested that necroptosis and ER stress were involved in the USP20-mediated response to I/R injury (**Figure 3A-B**). Subsequently, we investigated the relationship between USP20 and ER stress as well as necroptosis in myocardial injury induced by H/R in cardiomyocytes. Cardiomyocytes were subjected to transfection with either siRNA or USP20 plasmid to facilitate the knockdown or overexpression of USP20, respectively (**Figure S3A-D**). The results demonstrated that the expression levels of p-PERK, IRE1α and ATF6, p-MLKL, p-RIPK1 and p-RIPK3 were significantly elevated in cardiomyocytes subjected to H/R. Silencing USP20 expression resulted in a reduction in the expression of transmembrane stress sensors proteins localized to the ER (p-PERK, IRE1α and ATF6), while concurrently enhancing the expression of necroptosis-related proteins induced by H/R (**Figure 3C-F**). Conversely, overexpression of USP20 yielded opposite effects (**Figure 3G-J**). We next examined whether USP20 regulates oxidative stress during H/R injury. Exposure to H/R markedly promoted oxidative damage, characterized by excessive ROS accumulation, enhanced protein carbonylation, increased MDA and DHE levels, and impaired SOD activity. Notably, USP20 knockdown exacerbated the high levels of protein carbonylation, further intensifying the accumulation of MDA and DHE, while reducing the SOD activity induced by H/R (**Figure 3K-N**). In contrast, overexpression of USP20 mitigated the elevated levels of protein carbonylation, MDA and DHE, while restoring the inhibited SOD activity caused by H/R injury (**Figure 3O-R**).

### Identification of glucose-regulated protein 78 (GRP78) as a potential substrate for USP20 in myocardial injury induced by I/R

**Figure 4:**
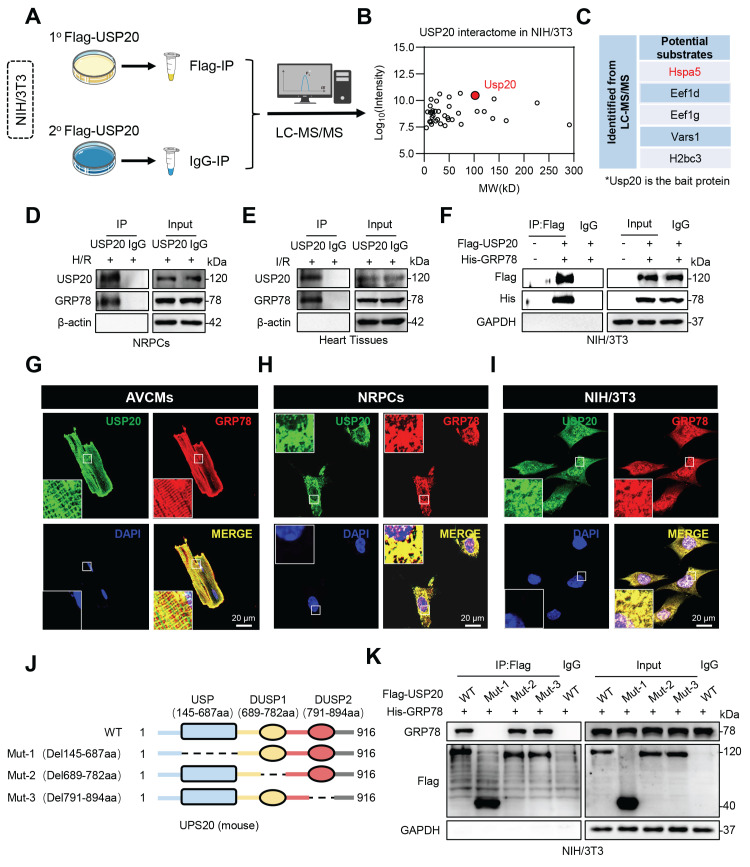
** Identification of Glucose-regulated Protein 78 (GRP78) as a Potential Substrate for USP20 in Myocardial Injury induced by I/R.** (**A**) The workflow for USP20 substrate screening. NRPCs were transfected with Flag-USP20 plasmids, and subsequently subjected to H/R stimulation. Anti-Flag antibodies and protein G-Sepharose beads were added to the cell samples for co-immunoprecipitation (Co-IP). The binding proteins were then extracted, digested into peptides, and analyzed using liquid chromatography tandem mass spectrometry (LC-MS/MS). (**B**) Two-dimensional (2D) plot with log_10_ signal intensity of the quantified proteins on the y axis (revealing the enrichment in Flag-USP20-IP) and molecular weight (MW) of protein on the x axis. (**C**) Table showed that the candidate substrates of USP20 screened by LC-MS/MS. (**D, E**) Co-IP of USP20 and GRP78 in NRPCs subjected to H/R (**D**) and in heart tissue of mice induced by I/R (**E**). Endogenous USP20 was immunoprecipitated by anti-USP20. (**F**) Co-IP of USP20 and GRP78 in NIH/3T3 cells co-transfected with plasmids encoding Flag–USP20 and His-GRP78. Exogenous USP20 was immunoprecipitated by anti-Flag antibody. (**G-I**) Colocalization of Flag-USP20 (green) and His-GRP78 (red) in H/R-challenged adult ventricular cardiomyocytes (AVCMs) (**G**), NRPCs (**H**) and NIH/3T3 cells (**I**). (**J**) Schematic illustration of the USP20 domain. (**K**) Co-IP of wt-USP20, mut-USP20 and GRP78 in NIH/3T3 cells co-transfected with plasmids of Flag-wt-USP20, Flag-mut-USP20 and His-GRP78. Exogenous normal or mutated USP20 was immunoprecipitated by anti-Flag antibody.

DUBs execute their functions through the modification of target proteins. To determine the substrate protein modulated by UPS20 during I/R, NRPCs were transfected to overexpress USP20 before exposure to H/R. Co-IP coupled with LC-MS/MS was utilized to comprehensively screen and identify candidate substrate proteins that physically interact with USP20 (**Figure 4A**). Among the 104 proteins identified via LC-MS/MS analysis, 37 substrate proteins showing over a twofold change were chosen as potential candidates (**Figure 4B**). In consideration of the potential for false positives in LC-MS/MS results, the experiment was replicated under identical conditions, resulting in the identification of 5 candidates, including Hspa5, Eef1d, Eef1g, Vars1 and H2bc3 (**Figure 4C**). Co-IP experiments were further performed to confirm potential interactions between USP20 and these candidate proteins in NRPCs during H/R exposure. The results showed that Hspa5 (also known as GRP78) and USP20 physically interact in NRPCs during H/R exposure (**Figure 4D**). In contrast, no detectable interaction between USP20 and the other four candidates (Eef1d, Eef1g, Vars1, or H2bc3) was observed under the same experimental conditions (**Figure S4**). The interaction between USP20 and GRP78 was further confirmed in myocardial tissues following I/R injury (**Figure 4E**). To further verify this association, NIH/3T3 cells were co-transfected with USP20 and GRP78 expression plasmids, followed by co-immunoprecipitation analysis (**Figure 4F**). In parallel, immunofluorescence staining demonstrated clear colocalization of USP20 and GRP78 in AVCMs, NRPCs, and NIH/3T3 cells (**Figure 4G-I**). Based on these findings, GRP78 was identified as a potential substrate protein of USP20 for subsequent mechanistic studies.

USP20 contains three major structural domains, including a ubiquitin-specific protease (USP) domain and two dual-specificity phosphatase domains, DUSP1 and DUSP2 (**Figure 4J**). Next, USP20 plasmids with mutations in various regions were generated, followed by Co-IP assays. The findings demonstrated that GRP78 interacted with USP20 via its USP domain (**Figure 4K**). These results suggest that GRP78 may be a USP20 substrate during I/R-induced myocardial injury.

### USP20 deubiquitinates GRP78 by cleaving K63-linked ubiquitin chains through its C154 active site

**Figure 5:**
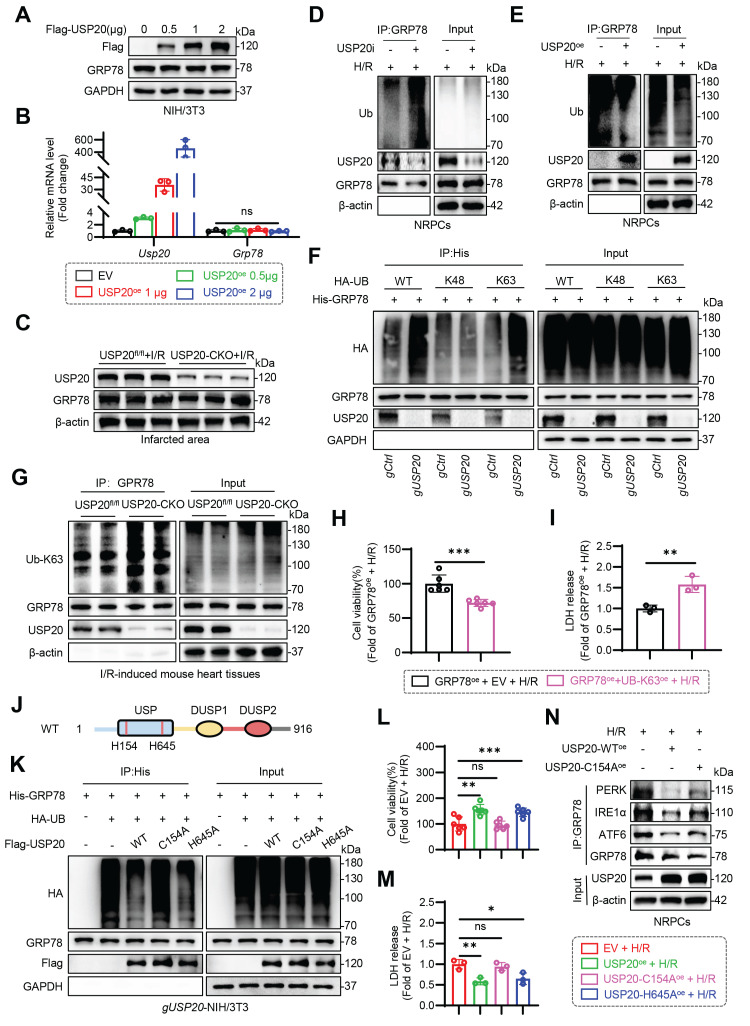
** USP20 Deubiquitinates GRP78 by Cleaving K63-linked Ubiquitin Chains Through Its C154 Active Site.** (**A**) The protein expression level of GRP78 in NIH/3T3 cells transfected with different doses of Flag-USP20 plasmids. n = 3. (**B**) The mRNA expression levels of *Usp20* and *Grp78* in NIH/3T3 cells transfected with different doses of Flag-USP20 plasmids. n = 3. (**C**) The protein expression of USP20 and GRP78 in infarcted regions of heart tissue from USP20^fl/fl^ mice and USP20-CKO mice subjected to I/R injury. n = 6. (**D**) Immunoprecipitation of GRP78 in USP20-silenced NRPCs subjected to H/R. (**E**) Immunoprecipitation of GRP78 in NRPCs overexpressing USP20 subjected to H/R. (**F**) Immunoprecipitation of GRP78 in *gCtrl* or *gUSP20* NIH/3T3 cells that co-transfected with plasmids encoding His-GRP78, HA-UB, HA-K48-UB and HA-K63-UB prior to treatment with MG132 (10 μM). The ubiquitinated form of GRP78 was detected through immunoblotting utilizing a His-specific antibody to clarify the ubiquitination patterns of GRP78 regulated by USP20. (**G**) Immunoprecipitation of GRP78 in I/R-induced heart tissues from USP20^fl/fl^ mice and USP20-CKO mice. Ubiquitinated GRP78 was assessed through immunoblotting using an UB-K63 antibody to elucidate the K63 ubiquitination level of GRP78 regulated by USP20. (**H, I**) Assessment of cell viability (**H**) and LDH release (**I**) in H/R-induced NRPCs overexpressing GRP78 and Ub-K63 plasmids. n = 3. (**J**) Schematic illustration of the active site of USP20. (**K**) Immunoprecipitation of GRP78 in *gUSP20* NIH/3T3 cells that co-transfected with plasmids encoding His-GRP78, HA-UB, USP20-WT, USP20-C154A and USP20-H645A plasmids prior to treatment with MG132 (10 μM). The ubiquitinated form of GRP78 was detected through immunoblotting utilizing a His-specific antibody to clarify the ubiquitination level of GRP78 regulated by the active site of USP20. (**L, M**) Evaluation of cell viability (**L**) and LDH release (**M**) in H/R-induced NRPCs overexpressing USP20-WT, USP20-C154A and USP20-H645A plasmids. n = 3. (**N**) Co-immunoprecipitation analysis showing the interaction of GRP78 with PERK, IRE1α, and ATF6 in NRPCs subjected to H/R after overexpression of USP20^WT^ or the catalytically inactive mutant USP20^C154A^. Data are expressed as the mean ± SD. ***, P < 0.001; **, P < 0.01; *, P < 0.05; ns, P > 0.05, ns: no differences. Student’s t-test for H and I; One-way ANOVA followed by Tukey's post hoc test for B, L and M.

DUBs exert their effects by deubiquitinating substrate proteins, thereby modulating the stability or functionality of these proteins. In the context of I/R treatment, neither overexpression nor cardiac-specific knockout of USP20 influenced GRP78 expression in NIH/3T3 cells or in mouse heart tissues, as illustrated in **Figure 5A-C and Figure S5A**. In addition, we did not find any effects of UPS20 deletion on the degradation rate of GRP78 in USP20 knockout (*gUsp20*) NIH/3T3 cells or wild-type (*gCtrl*) NIH/3T3 cells treated with cycloheximide (CHX) (**Figure S5B-C**). Similarly, no significant effect of USP20 on the degradation rate of GRP78 was observed in NIH/3T3 cells overexpressing USP20 (**Figure S5D-E**). Therefore, whether USP20 mediates the ubiquitination modification of GRP78 to regulate its function was next investigated. Interestingly, our research revealed that GPR78 could be modified through ubiquitination (**Figure S5F**).

Furthermore, we discovered that the inhibition of USP20 expression notably increased the ubiquitination levels of GRP78 (**Figure 5D**), whereas USP20 overexpression reduced it (**Figure 5E**). These suggest that USP20 might regulate the function of GRP78 through its deubiquitination activity. Subsequently, the particular ubiquitin (Ub) linkage types present in the polyubiquitin chains linked to GRP78 that are targeted by USP20 was investigated. The K48- and K63-linked ubiquitin chains are the most extensively studied forms 18-21. We therefore focused on K48- and K63-linked ubiquitin chains. Loss of USP20 significantly enhanced ubiquitin conjugation to GRP78 in the presence of Ub-WT or Ub-K63 constructs, whereas this effect was not observed in cells expressing the Ub-K48 mutant (**Figure 5F**). The outcome was corroborated in an animal model subject to I/R induction, suggesting that USP20 selectively removed K63-linked ubiquitination from GRP78 protein (**Figure 5G and Figure S5G**). Whether GRP78 ubiquitination has an impact on cell viability was subsequently investigated, and GRP78 was overexpressed in NRPCs (**Figure S6A-B**). The results demonstrated that the upregulation of GRP78 ubiquitination through UB-K63 plasmid overexpression significantly exacerbated H/R-induced cell injury (**Figure 5H-I**), indicating that GRP78 ubiquitination was involved in I/R-mediated myocardial injury.

The catalytic motifs C154 (cysteine at site 154) in USP20 have been identified as crucial for its deubiquitinating activity 22. Additionally, H645 (histidine at site 645) may also represent a potential functional site for the volatile deubiquitination of USP20 (**Figure 5J**). To examine their roles, C154A and H645A USP20 mutants were generated. Co-IP assays showed that USP20C154A had markedly reduced ability to remove ubiquitin from GRP78 compared with USP20H645A (**Figure 5K**), although both mutants retained GRP78-binding capacity (**Figure S7A-B**). Moreover, compared to the group overexpressing wild-type USP20, overexpression of USP20^C154A^ failed to reduce H/R-induced cell death and LDH release (**Figure 5L-M**). Notably, Co-IP analysis further showed that USP20^WT^ markedly reduced the association of GRP78 with PERK, IRE1α, and ATF6 under H/R conditions, whereas USP20^C154A^ failed to reduce these interactions (**Figure 5N and Figure S7C**). Collectively, these results demonstrate that USP20 deubiquitinates GRP78 through its C154 active site, thereby facilitating GRP78 activation, its dissociation from ER stress sensors, and subsequent cardioprotective signaling.

### USP20 deubiquitinates GRP78 at the K602 residue

**Figure 6:**
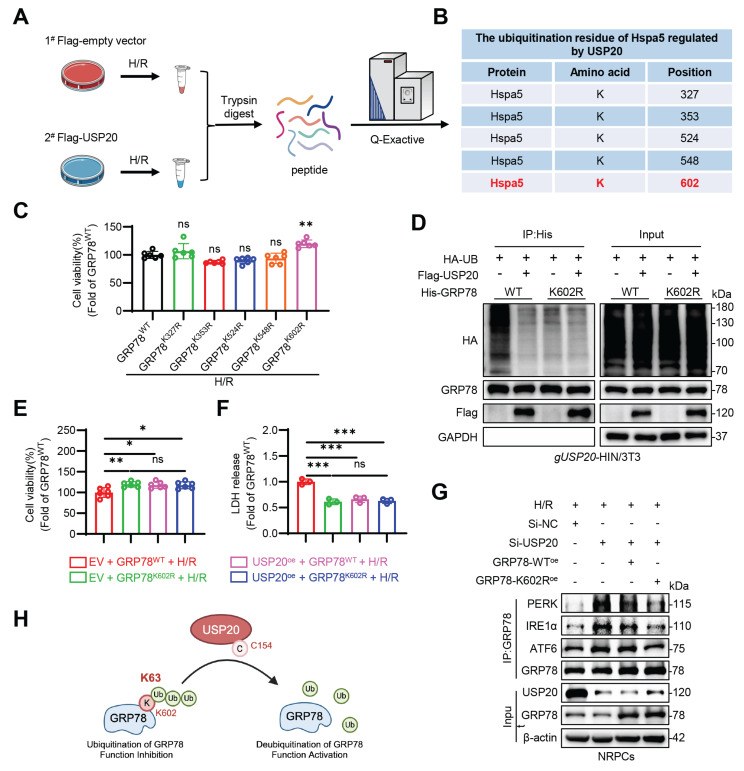
** USP20 Deubiquitinates GRP78 at the K602 Residue.** (**A**) Schematic illustration of a Co-IP-based ubiquitinated peptide enrichment analysis for screening ubiquitination lysine residues on GRP78 regulated by USP20. (**B**) Table showed that ubiquitination lysine residues on GRP78, identified from ubiquitinome analysis. (**C**) Evaluation of cell viability in H/R-induced NRPCs overexpressing plasmids with mutations at various sites of GRP78, as well as the wild-type GRP78. (**D**) Immunoprecipitation of GRP78 in *gUSP20* NIH/3T3 cells that co-transfected with plasmids encoding Flag-USP20, His-GRP78-WT, His-GRP78-K602R and HA-UB plasmids prior to treatment with MG132 (10 μM). Ubiquitinated GRP78 was assessed through immunoblotting utilizing a His-specific antibody to clarify the ubiquitination residue of GRP78 regulated by the USP20. (**E, F**) Assessment of cell viability (**E**) and LDH release (**F**) in H/R-induced NRPCs overexpressing Flag-USP20, His-GRP78-WT, His-GRP78-K602R plasmids. n = 3. Co-immunoprecipitation analysis of the interaction between GRP78 and PERK, IRE1α, and ATF6 in H/R-treated NRPCs following USP20 knockdown and GRP78^WT^ or GRP78^K602R^ overexpression. (**H**) Schematic illustrating that USP20 activates the GRP78 via deubiquitinating GRP78 at residue K602 by its active site C154. Data are expressed as the mean ± SD. ***, P < 0.001; **, P < 0.01; *, P < 0.05; ns, P > 0.05, ns: no differences. One-way ANOVA followed by Tukey's post hoc test for C, E and F.

To elucidate the specific site of GRP78 deubiquitination regulated by USP20, an enrichment analysis of ubiquitinated peptides using Co-IP-based method was conducted in HL-1 cells overexpressing USP20 (**Figure 6A**). The analysis identified five potential lysine residues subject to ubiquitination with the GRP78 protein: K327, K353, K524, K548 and K602 (**Figure 6B**). Subsequently, five mutants of GRP78 were constructed through the substitution of lysine residues with arginine. Of all GRP78 ubiquitination-site mutants examined, only GRP78^K602R^ conferred enhanced resistance to H/R-induced injury in HL-1 cells relative to GRP78^WT^, highlighting K602 as a potentially critical residue for functional ubiquitination (**Figure 6C**). Further Co-IP experiments confirmed that USP20 significantly diminished the ubiquitination level of the GRP78^WT^ protein. When subjected to mutation at the K602 site, there was a marked decrease in ubiquitination levels of the GRP78 protein (**Figure S8A**). Notably, USP20 did not induce any additional decrease in its ubiquitination level (**Figure 6D**). Subsequent *in vitro* experiments further corroborated these findings. In comparison to cells overexpressing the GRP78^WT^ protein, those containing GRP78^K602R^ protein exhibited substantially improved cell viability. Further investigations revealed that under H/R stimulation conditions, co-transfection of cardiomyocytes with plasmids overexpressing USP20 together with either GRP78^WT^ or GRP78^K602R^ led to significant improvements in cell viability relative to cardiomyocytes overexpressing GRP78^WT^ alone. However, in H/R-induced cardiomyocytes overexpressing GRP78^K602R^, no differences in cardiomyocyte viability or LDH release were observed between groups, irrespective of whether USP20 was co-overexpressed (**Figure 6E-F**). Co-IP analysis further revealed that USP20 knockdown promoted the retention of GRP78 on PERK, IRE1α, and ATF6 under H/R conditions. Reintroduction of GRP78^WT^ produced only a limited effect on this enhanced sensor engagement, whereas GRP78^K602R^ markedly weakened GRP78 binding to these ER stress sensors (**Figure 6G** and **Figure S8B**). In addition, overexpression of GRP78^K602R^ significantly enhanced cell viability compared with GRP78^WT^ overexpression in USP20-knockdown cardiomyocytes subjected to H/R injury (**Figure S8C-D**). Together, these results identify K602 as a functionally important ubiquitination site that determines the activation state of GRP78 and its ability to transmit USP20-dependent cytoprotective signaling (**Figure 6H**).

### USP20 protects myocardial tissue from I/R injury in a GRP78-dependent manner

**Figure 7:**
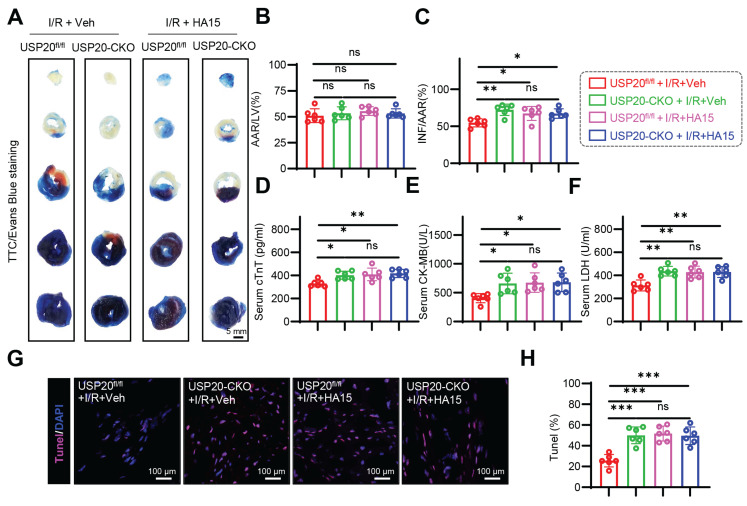
** USP20 Protects Myocardial Tissue From I/R Injury in a GRP78-dependent Manner.** After 24 h of treatment with the GRP78 inhibitor HA15 (15 mg/kg) in both USP20^fl/fl^ mice and USP20-CKO mice, the mice were subjected to a 30 min ischemic event, followed by a subsequent period of 24 h of reperfusion. This procedure was conducted to induce I/R injury. (**A-C**) Representative images of TTC/Evans Blue staining of heart tissue (**A**) and the statistical results of the ratio of AAR to left ventricular area (**B**) and INF to AAR (**C**). Scale bar 50 mm. (**D-F**) Serum concentrations of cTnT (**D**), CK-MB (**E**), and LDH (**F**) in each group. (**G, H**) Representative images of TUNEL staining of myocardial tissue in each group (**G**) and the statistical results (**H**). Data are expressed as the mean ± SD. ***, P < 0.001; **, P < 0.01; *, P < 0.05; ns, P > 0.05, ns: no differences. n = 6. One-way ANOVA followed by Tukey's post hoc test for B, C, D, E, F and H.

Further *in vivo* experiments using the GRP78 inhibitor HA15 verified the substrate protein through which USP20 exerts its function during I/R. At the dose used here, HA15 did not impair cardiac function or increase serum cTnT, CK-MB, or LDH. H&E staining also revealed no overt pathological injury in the brain, liver, kidney, lung, or heart (**Figure S9A-G**). Compared with I/R-treated USP20^fl/fl^ mice, HA15-treated mice showed larger infarct areas. However, HA15 did not further enlarge infarct size in I/R-treated USP20-CKO mice (**Figure 7A-C**). Consistently, HA15 did not further increase cTnT, CK-MB, LDH, or TUNEL-positive cells in USP20-CKO mice subjected to I/R (**Figure 7D-H**). Furthermore, we effectively achieved the knockdown of GPR78 expression in cardiomyocytes (**Figure S10A-B**). It was observed that in cardiomyocytes with low levels of GRP78, the overexpression of USP20 failed to promote protein carbonylation or alter the levels of MDA, SOD, and DHE (**Figure S10C-F**). Simultaneously, we noted that there were no significant alterations in the phosphorylation levels of MLKL, RIPK1, and RIPK3 under these experimental conditions (**Figure S10G-H**). Consistently, CCK-8 and LDH release assays showed that USP20 overexpression improved cell viability and reduced LDH release under H/R conditions, whereas these protective effects were lost after GRP78 knockdown (**Figure S10I-J**). These results provide robust evidence that USP20 regulates I/R injury in a GRP78-dependent manner.

### Overexpression of USP20 ameliorates myocardial injury induced by I/R

**Figure 8:**
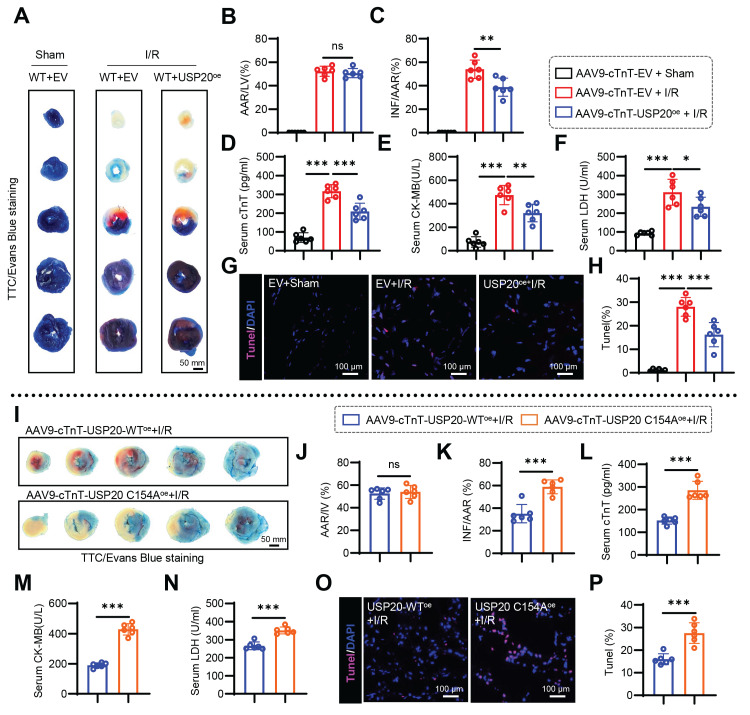
** Overexpression of USP20 Ameliorates Myocardial Injury induced by I/R.** After the specific overexpression of USP20 in mouse cardiomyocytes through the administration of AAV9-cTnT-USP20^oe^ virus or AAV9-cTnT-USP20-C154A^oe^ virus in wild-type mice, along with the use of AAV9-cTnT-Empty Vector for one month, the mice were subjected to a 30 min ischemic event, followed by a subsequent period of 24 h of reperfusion. This procedure was conducted to induce I/R injury. (**A-C**) Representative images of TTC/Evans Blue staining of heart tissue (**A**) and the statistical results of the ratio of AAR to left ventricular area (**B**) and INF to AAR (**C**). Scale bar 50 mm. (**D-F**) Serum levels of cTnT (**D**), CK-MB (**E**), and LDH (**F**) in each group. (**G, H**) Representative images of TUNEL staining of myocardial tissue in each group (**G**) and the statistical results (**H**). (**I-K**) Representative images of TTC/Evans Blue staining of heart tissue (**I**) and the statistical results of the ratio of AAR to left ventricular area (**J**) and INF to AAR (**K**) in in mice with cardiac-specific overexpression of USP20 versus those with cardiac-specific overexpression of USP20^C154A^. Scale bar 50 mm. (**L-N**) Serum levels of cTnT (**L**), CK-MB (**M**), and LDH (**N**) in mice with cardiac-specific overexpression of USP20 versus those with cardiac-specific overexpression of USP20^C154A^. (**O, P**) Representative images of TUNEL staining of myocardial tissue in mice with cardiac-specific overexpression of USP20 versus those with cardiac-specific overexpression of USP20^C154A^ (**O**) and the statistical results (**P**). Data are expressed as the mean ± SD. ***, P < 0.001; **, P < 0.01; *, P < 0.05; ns, P > 0.05, ns: no differences. n = 6. One-way ANOVA followed by Tukey's post hoc test for B, C, D, E, F, H, J, K, L, M, N and P.

AAV9, which specifically overexpresses USP20 in cardiomyocytes, was successfully constructed to achieve targeted expression of USP20 in cardiac tissue of mice via tail vein injection. Cardiomyocyte-specific transduction and efficient USP20 overexpression mediated by AAV9 were confirmed by Western blot analysis (**Figure S11A**), and hepatic and renal function did not differ between control and USP20-overexpressing mice (**Figure S11B-D**). We then assessed the impact of USP20 overexpression during myocardial I/R injury. Compared with AAV9-EV, AAV9-USP20 substantially reduced infarct size after I/R (**Figure 8A-C**), decreased serum cTnT, CK-MB, and LDH (**Figure 8D-F**), and lowered myocardial TUNEL-positive cells (**Figure 8G-H**). However, the protective effect conferred by USP20 overexpression is abolished upon mutation of the C154 residue of USP20 (**Figure 8I-P and Figure S11E**). These results demonstrate that USP20 mitigates myocardial I/R injury in cardiomyocytes and that C154 is essential for its function.

## Discussion

Myocardial I/R injury is a major contributor to poor outcomes in cardiovascular disease, including coronary heart disease, and involves multiple dysregulated molecular networks. This study defined the function and mechanism of USP20 in myocardial I/R injury. We found that USP20 was reduced in I/R-injured myocardium and H/R-treated cardiomyocytes. USP20-CKO worsened myocardial injury, remodeling, and dysfunction after I/R, whereas cardiomyocyte-specific USP20 overexpression was protective. Mechanistically, USP20 interacted with GRP78 in H/R-treated cardiomyocytes and removed K63-linked ubiquitin chains from GRP78 at K602 through its C154 active site. This deubiquitination promoted GRP78 activation and dissociation from ER stress sensors, thereby facilitating adaptive ER stress signaling and ultimately alleviating myocardial I/R injury.

Ubiquitination is a post-translational modification that controls diverse protein functions. Deubiquitination reverses this process by removing ubiquitin from substrates, thereby influencing protein degradation, signaling, and activity 23. In recent years, scholarly research has shown that deubiquitinases serve essential regulatory functions in myocardial I/R injury by targeting different substrate proteins and affecting multiple signaling pathways 24-27. In the present study, we found that USP20 was significantly downregulated in myocardium tissues subjected to I/R. The deficiency of USP20 aggravated myocardial injury and compromised cardiac function resulting from I/R, whereas overexpression of USP20 ameliorated these effects. These bidirectional loss- and gain-of-function data identify USP20 as an important endogenous protective factor against myocardial I/R injury and support the therapeutic potential of targeting DUB-mediated regulatory pathways in ischemic heart disease.

At the mechanistic level, this study has, for the first time, identified GRP78 as the molecular target of USP20. GRP78, also referred to as heat-shock protein 5 (HSPA5), serves as a primary molecular chaperone within the ER and functions as a fundamental regulator of the unfolded protein response (UPR), which is intricately linked to ER stress 28, 29. GRP78 participates in protein folding and assembly, degradation of misfolded proteins, maintenance of endoplasmic reticulum calcium homeostasis and regulation of ER stress sensor activation 30. Under normal physiological conditions, GRP78 binds to transmembrane stress sensors on the ER membrane such as PERK, IRE1, and ATF6, keeping them in an inactive state. This interaction facilitates proper folding of newly synthesized proteins while preventing premature initiation of the UPR 31, 32. When there is an excessive accumulation of unfolded or misfolded proteins in the ER, GRP78 becomes activated and dissociates from these transmembrane sensors. As a results, PERK, IRE1 and ATF6 are activated to initiate UPR signaling 33. In this manner, GRP78 contributes to restoring normal ER function and preserving ER homeostasis.

During myocardial I/R, ER function is disrupted by ROS generation and calcium imbalance, leading to accumulation of unfolded or misfolded proteins 34-36. In response to this situation, GRP78 is activated and dissociates from ER transmembrane proteins, thereby initiating the UPR to alleviate the burden of these improperly folded proteins. This process not only aids in re-establishing ER homeostasis, but also mitigates oxidative stress and protects cardiomyocytes from I/R injury. It has been documented that GRP78 undergoes modification by polyubiquitination through the UPS to fulfill its functions 37-39. OTUD3 has been reported to interact with GRP78. OTUD3 deubiquitylates and stabilizes GRP78, promoting lung cancer cell proliferation and tumorigenesis 40. Targeting the OTUD3-GRP78 axis presents a promising therapeutic avenue for lung cancer 41. In our current investigation, we observed that GRP78 was deubiquitinated by USP20 under the conditions of myocardial I/R injury. At the mechanistic level, USP20 was found to catalyze the removal of K63-linked ubiquitin chains from GRP78 at the K602 residue via its C154 catalytic domain, thereby regulating GRP78 functional activation and suppressing maladaptive ER stress during myocardial I/R injury. Notably, this study identified both the precise ubiquitination site on GRP78 and the specific ubiquitin linkage type regulated by USP20. The ubiquitination of K63 linkages is generally recognized as a non-degradative modification that modulates protein activity, conformation, localization, and protein-protein interactions^42^. Therefore, the USP20-mediated deubiquitination of GRP78 is very likely to act as a molecular “switch”, directly altering the conformation of GRP78 or its interaction efficiency with stress sensors, thereby transforming it from a relatively inactive state to an activated state, and enhancing its ability to cope with the misfolded proteins. This discovery has deepened the understanding of the refined regulation of GRP78 functions.

As the core of negative feedback regulation of ER stress, the activation of GRP78 can effectively alleviate stress signals and inhibit the excessive activation of pro-apoptotic pathways. The present results showed that elevated USP20 expression enhanced adaptive ER stress signaling and attenuated necroptotic activation, which is consistent with the functional activation of GRP78. In addition, ER stress and oxidative stress exhibit a mutually causal relationship in I/R injury, forming a vicious cycle. The activation of GRP78 contributes to the restoration of the redox homeostasis of the endoplasmic reticulum and diminishes the production of reactive oxygen species. Meanwhile, the alleviation of oxidative stress is also beneficial for maintaining the protein folding environment. Thus, USP20 may simultaneously improve ER stress, oxidative stress, and necroptosis by regulating GRP78.

These findings have translational implications. AAV9-mediated USP20 overexpression reduced myocardial I/R injury, infarct size, cardiac dysfunction, and adverse remodeling. Together with the aggravated phenotype observed in cardiac-specific USP20 knockout mice, these results support a protective role of USP20 in myocardial I/R injury. In the present study, USP20 was overexpressed in mouse cardiac tissue using AAV9. We further evaluated hepatic and renal function after USP20 overexpression and observed no significant differences compared with the control group, suggesting that cardiac USP20 overexpression did not produce obvious hepatic or renal toxicity under our experimental conditions. Nevertheless, this study used a single viral dose to establish the gain-of-function model, and a formal dose-response analysis was not performed. Future studies should evaluate multiple AAV9-USP20 doses to define the therapeutic window, dose-efficacy relationship, and long-term safety profile of USP20-based gene therapy.

Despite the therapeutic promise of targeting USP20, several challenges remain. The acute nature of myocardial I/R injury and the requirement for cardiac-specific delivery pose major obstacles to clinical translation. Although cardiac-targeted AAV9-mediated gene delivery is effective in preclinical models, its clinical application may be limited by immunogenicity, potential off-target effects, and long-lasting expression. Therefore, small-molecule activators of USP20 may represent a more flexible and clinically feasible therapeutic strategy. Such molecules could potentially be conjugated to cardiac-targeting peptides or encapsulated in lipid nanoparticles to enhance myocardial delivery. For post-reperfusion therapy in acute myocardial infarction, local delivery through interventional approaches, such as intracoronary administration, may provide a direct strategy to achieve high myocardial drug exposure while minimizing systemic toxicity.

In summary, this study identifies an unrecognized USP20-GRP78 axis in myocardial I/R injury. USP20 removes K63-linked ubiquitin chains from GRP78 at K602, promotes GRP78 activation, facilitates adaptive ER stress signaling, and suppresses necroptosis, thereby protecting the ischemic/reperfused heart. These findings deepen our understanding of non-degradative ubiquitin regulation of GRP78 and support the development of USP20-based strategies for myocardial I/R injury.

## Conclusions

USP20 plays a critical role in myocardial I/R injury. By interacting with GRP78, USP20 removes K63-linked ubiquitin chains from GRP78 at K602 through its C154 active site. This modification activates GRP78, enhances adaptive ER stress responses, and reduces I/R-induced myocardial injury. The USP20-GRP78 axis therefore provides mechanistic insight into myocardial I/R injury and suggests USP20 as a potential therapeutic target for cardiac protection.

## Supplementary Material

Supplementary figures and tables.

## Data Availability

All data are included in the article and Supplementary Information or are available from the corresponding author upon reasonable request.

## Abbreviations

AAR: area at risk
ANP: Atrial Natriuretic Peptide
CK-MB: Creatine Kinase-MB Isoenzyme
cTnT: cardiac troponin T
DHE: dihydroethidium
DUBs: deubiquitinating enzymes
EF: ejection fraction
ER: endoplasmic reticulum
FS: fractional shortening
GRP78: glucose-regulated protein 78
H/R: hypoxia/reoxygenation
INF: infarcted size
IP: immunoprecipitation
I/R: ischemia/reperfusion
LAD: left anterior descending
LDH: lactate dehydrogenase
MDA: activity and malondialdehyde
ROS: reactive oxygen species
SOD: superoxide dismutase
Ub: ubiquitin
USP20: ubiquitin-specific peptidase 20
UPR: unfolded protein response
UPS: ubiquitin-proteasome system
