# Supplementary material for: Cardiomyocyte-derived USP20 mitigates myocardial ischemia/reperfusion injury through deubiquitinating GRP78
Source: Theranostics. 2026 Jun 17;16(13):7594–612. doi: 10.7150/thno.132067 (PMC13295839; doi:10.7150/thno.132067)
Supplement: Supplementary file 1 — Supplementary figures and tables. [file thnov16p7594s1.pdf]

Cardiomyocyte-derived USP20 mitigates myocardial ischemia/reperfusion injury through deubiquitinating GRP78

Supplementary Information

Supplementary Information Contents:

1. Supplementary Figure S1-11
2. Supplementary Table S1-2

Supplementary Figures:

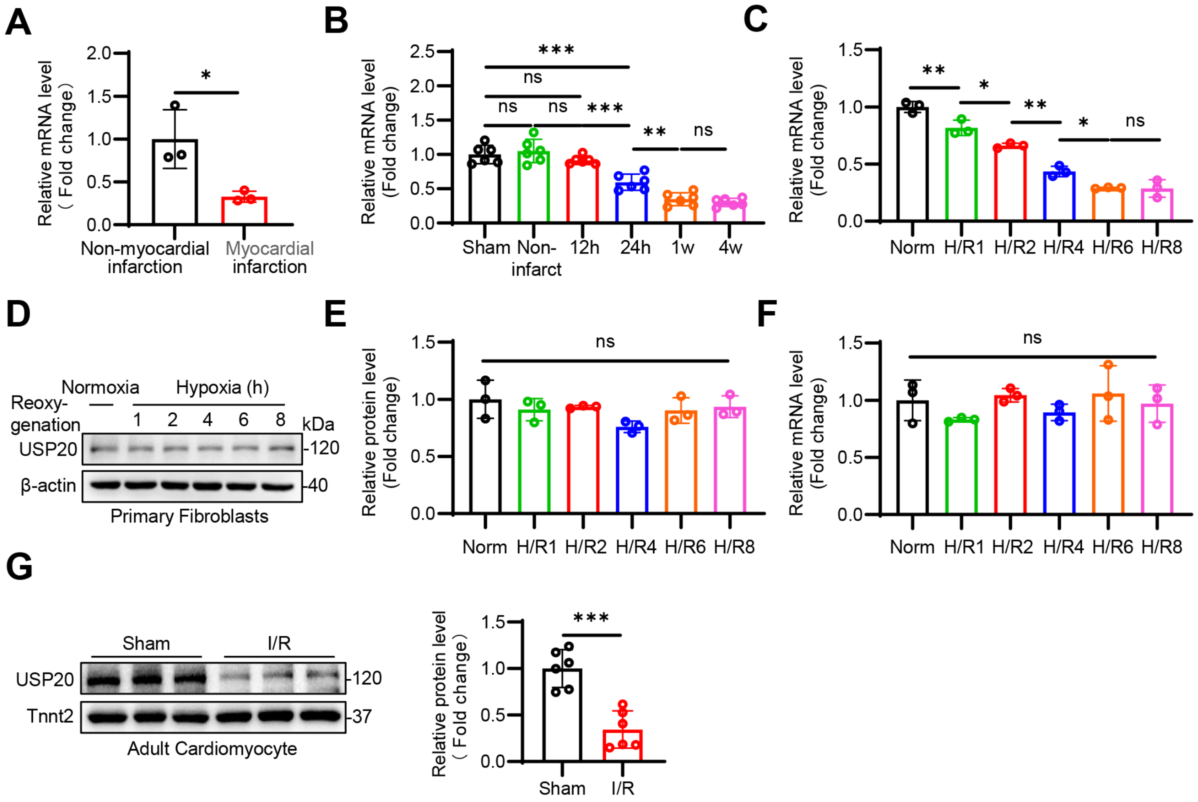

Supplementary Figure S1

(**A**) The mRNA expression levels of *Usp20* in both patients with myocardial infarction and control samples. n = 3.

(**B**) The mRNA levels of *USP20* in mouse heart tissue from the sham group and the I/R group in both non-infarcted and myocardial injury regions at various reperfusion time points. n = 6.

(**C**) The mRNA levels of *USP20* in NRPCs subjected to normoxia and hypoxia treatments followed by different reoxygenation time points. n = 3.

(**D, E**) The protein expression levels of USP20 in primary fibroblasts subjected to normoxia and hypoxia treatments followed by different reoxygenation time points evaluated by western blot (**D**) and the statistical results (**E**). n = 3.

(**F**) The mRNA levels of *USP20* in primary fibroblasts subjected to normoxia and hypoxia treatments followed by different reoxygenation time points. n = 3.

(**G**) Representative western blot for USP20 in adult cardiomyocytes isolated from sham or MI/RI mice and the statistical results. n = 3.

Data are expressed as the mean ± standard deviation (SD). ***, $P < 0.001$; **, $P < 0.01$; *, $P < 0.05$； ns, $P > 0.05$. ns: no differences. Student’s t-test for A and G; One-way ANOVA followed by Tukey's post hoc test for B, C, E and F.

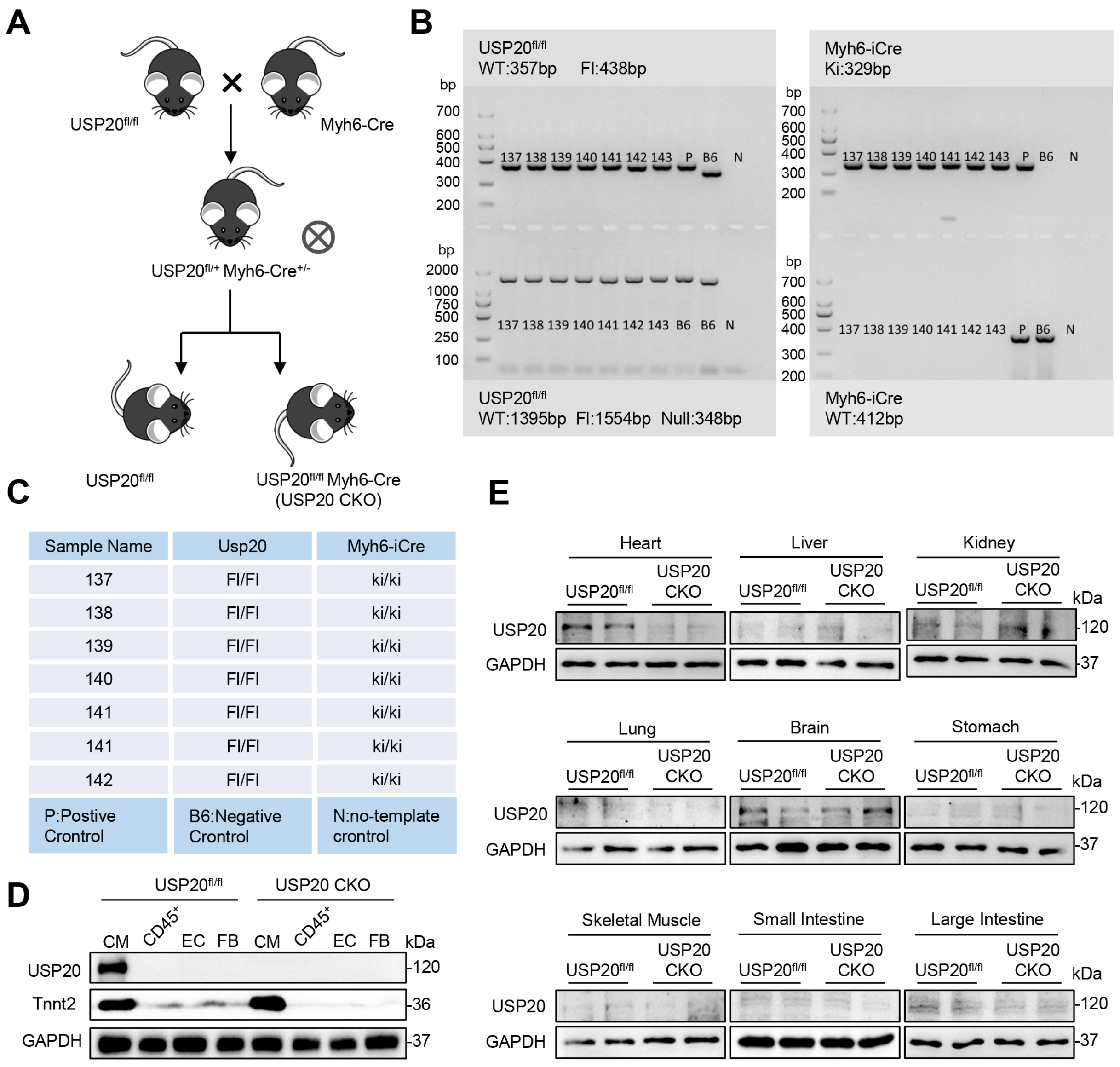

Sample Name	Usp20	Myh6-iCre
137	Fl/Fl	ki/ki
138	Fl/Fl	ki/ki
139	Fl/Fl	ki/ki
140	Fl/Fl	ki/ki
141	Fl/Fl	ki/ki
141	Fl/Fl	ki/ki
142	Fl/Fl	ki/ki
P:Postive Crontrol	B6:Negative Crontrol	N:no-template crontrol

Supplementary Figure S2

(**A**) Schematic diagram of the process for constructing USP20 cardiomyocyte specific knockout mice (USP20-CKO mice).

(**B**) The primers of *Usp20* (WT:357bp, FI:438bp) and *Myh6*-Cre (WT:412bp, KI:329bp) were respectively used for PCR to identify the genotype of mice. (P: Positive control; B6: Negative control; N: No-template control).

(**C**) Table of USP20 myocardial specific knockout mouse identification results.

(**D**) Representative western blot of USP20 in isolated cardiomyocytes (CM), CD45$^+$ cells, Endothelial cells (EC) and fibroblasts (FB) of USP20$^{fl/fl}$ and USP20-CKO mice.

(**E**) Representative western blot of USP20 in heart, liver, kidney, lung, brain, stomach, skeletal muscle, small intestine and large intestine tissues of USP20$^{fl/fl}$ and USP20-CKO mice.

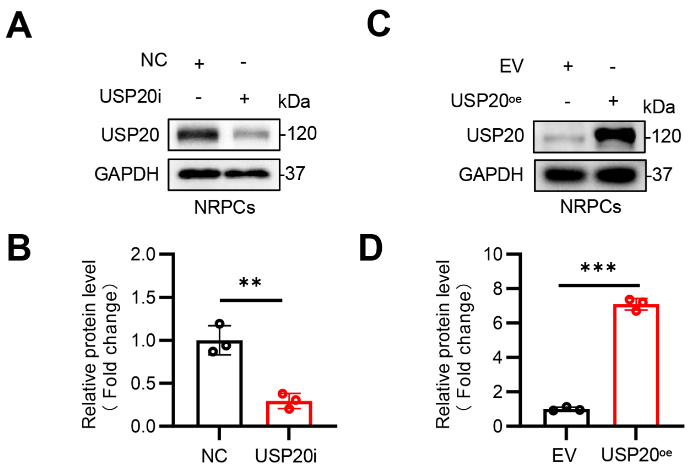

Supplementary Figure S3

(**A, B**) The protein expression level of USP20 in NRPCs following siRNA-mediated silencing of USP20 (**A**) and the statistical results (**B**). n = 3.

(**C, D**) The protein expression level of USP20 in NRPCs overexpressing USP20 via plasmid transfection (**C**) and the statistical results (**D**). n = 3.

Data are expressed as the mean ± SD. ***, P < 0.001; **, P < 0.01. Student's t-test for B and D.

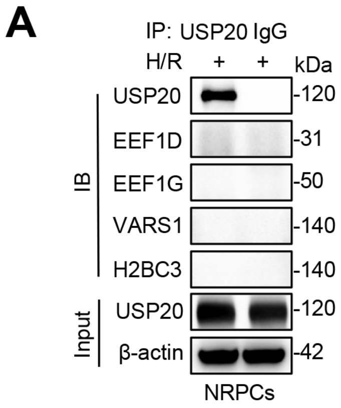

Supplementary Figure S4

(**A**) Co-IP of USP20, EEF1D, EEF1G, VARS1 and H2BC3 in NRPCs subjected to H/R. Endogenous USP20 was immunoprecipitated by anti-USP20.

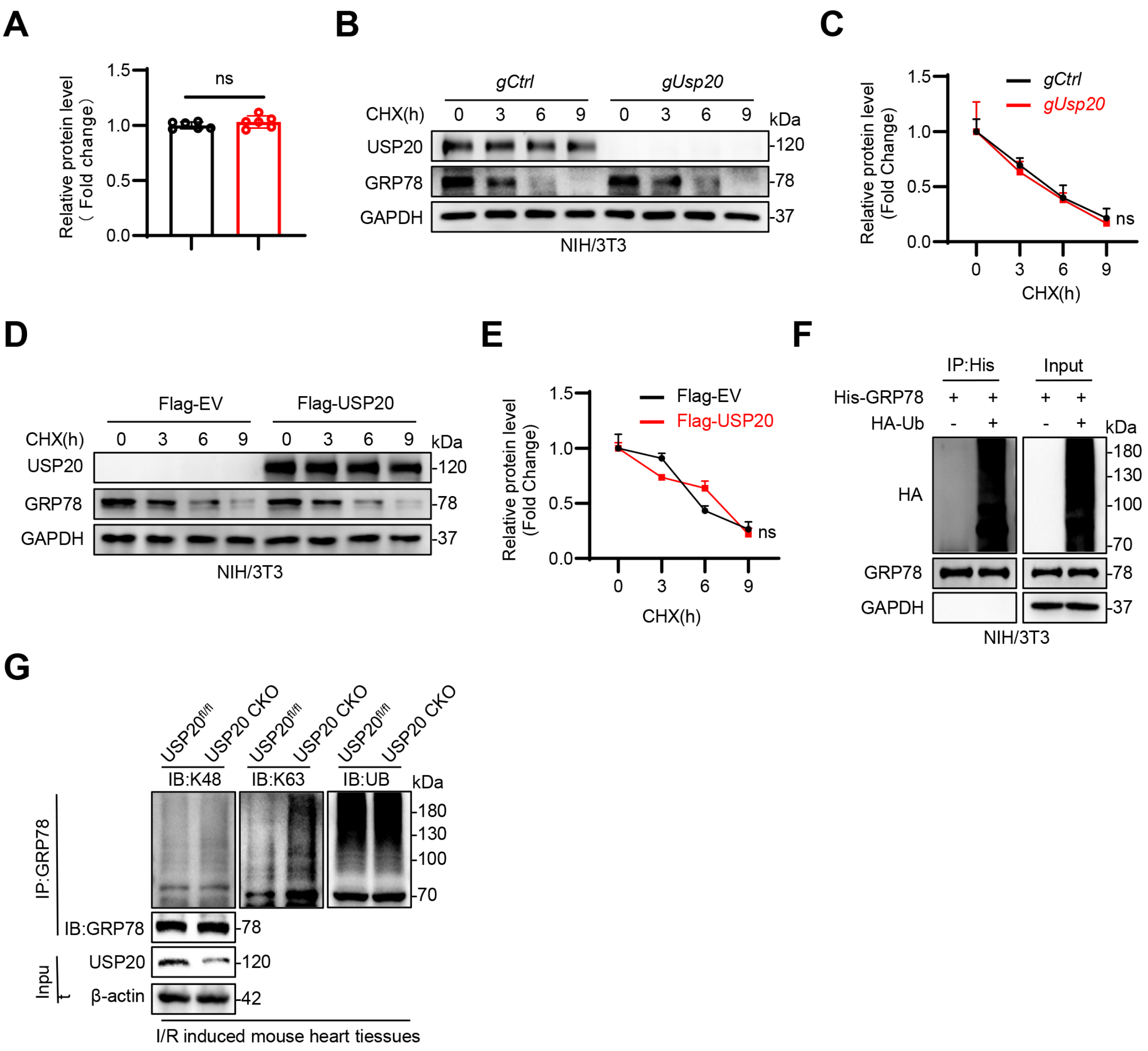

Supplementary Figure S5

(**A**) The corresponding quantitative data of **Figure 5C**.

(**B, C**) The protein expression levels of USP20 and GRP78 in *gCtrl* or *gUSP20* NIH/3T3 cells (**B**) and the statistical results (**C**). n = 3.

(**D, E**) The protein expression levels of USP20 and GRP78 in Flag-EV or Flag-USP20 NIH/3T3 cells (**D**) and the statistical results (**E**). n = 3.

(**F**) Immunoprecipitation of GRP78 in NIH/3T3 cells that co-transfected with plasmids encoding His-GRP78 and HA-UB.

(**G**) Immunoprecipitation of GRP78 in I/R induced mouse heart tissues from USP20$^{fl/fl}$ or USP20-CKO. The ubiquitinated form of GRP78 was detected through immunoblotting utilizing UB antibody, UB-K48 antibody and UB-K63 antibody to clarify the ubiquitination patterns of GRP78 regulated by USP20.

Data are expressed as the mean ± SD. ns, P > 0.05, ns: no differences. EV: empty vector. One-way ANOVA followed by Tukey post-hoc tests for C and E.

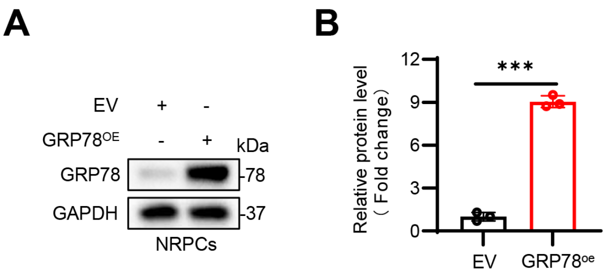

Supplementary Figure S6

(**A, B**) The protein expression level of GRP78 in NRPCs overexpressing GRP78 via plasmid transfection (**A**) and the statistical results (**B**). n = 3.

Data are expressed as the mean ± SD. ***, $P < 0.001$. Student's t-test for B.

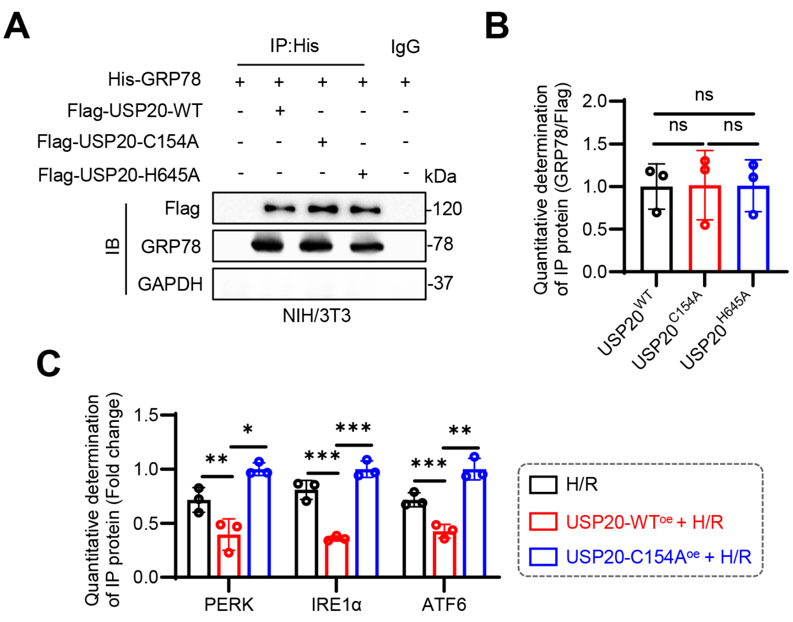

Supplementary Figure S7

(A-B) Immunoprecipitation of GRP78 in NIH/3T3 cells that co-transfected with plasmids encoding His-GRP78, Flag-USP20-C154A and Flag-USP20-H645A (**A**) and the statistical results (**B**). n = 3.

(C) The the statistical results of PERK/ GRP78, IRE1α/GRP78 and ATF6/GRP78 band intensity ratios in IP assays of **Figure 5N**. n=3

Data are expressed as the mean ± SD. *, P < 0.05; **, P < 0.01; ***, P < 0.001; ns, P > 0.05. ns: no differences. One-way ANOVA followed by Tukey's post hoc test for B, and C.

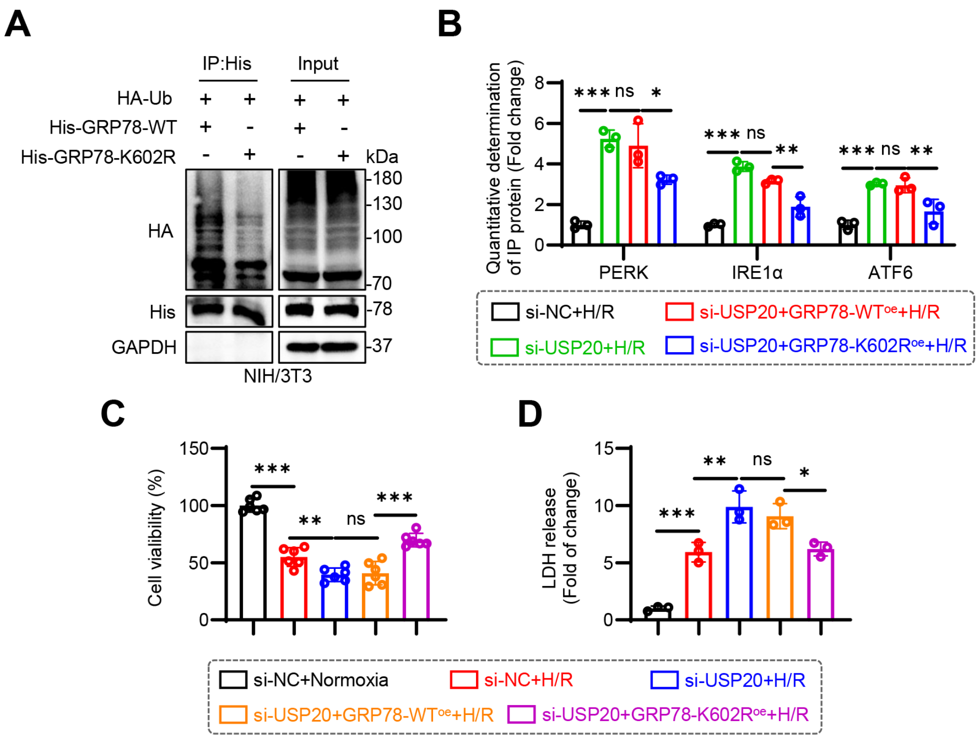

Supplementary Figure S8

(**A)** Immunoprecipitation of GRP78 in NIH/3T3 cells that co-transfected with plasmids encoding His-GRP78-WT, His-GRP78-K602R and HA-UB prior to treatment with MG132 (10 μM). The ubiquitination level of GRP78 was detected through immunoblotting utilizing a HA-specific antibody.

(B) The the statistical results of PERK/ GRP78, IRE1α/GRP78 and ATF6/GRP78 band intensity ratios in IP assays of **Figure 6G**. n=3.

(C-D) CCK8 assay (C) and LDH release assay (D) showing the effects of $GRP78^{WT}$ or $GRP78^{K602R}$ overexpression on cell viability and cellular injury in USP20-deficient NRPCs subjected to H/R treatment.

Data are expressed as the mean ± SD. *, P < 0.05; **, P < 0.01; ***, P < 0.001; ns, P > 0.05. ns: no differences. One-way ANOVA followed by Tukey's post hoc test for B, C and D.

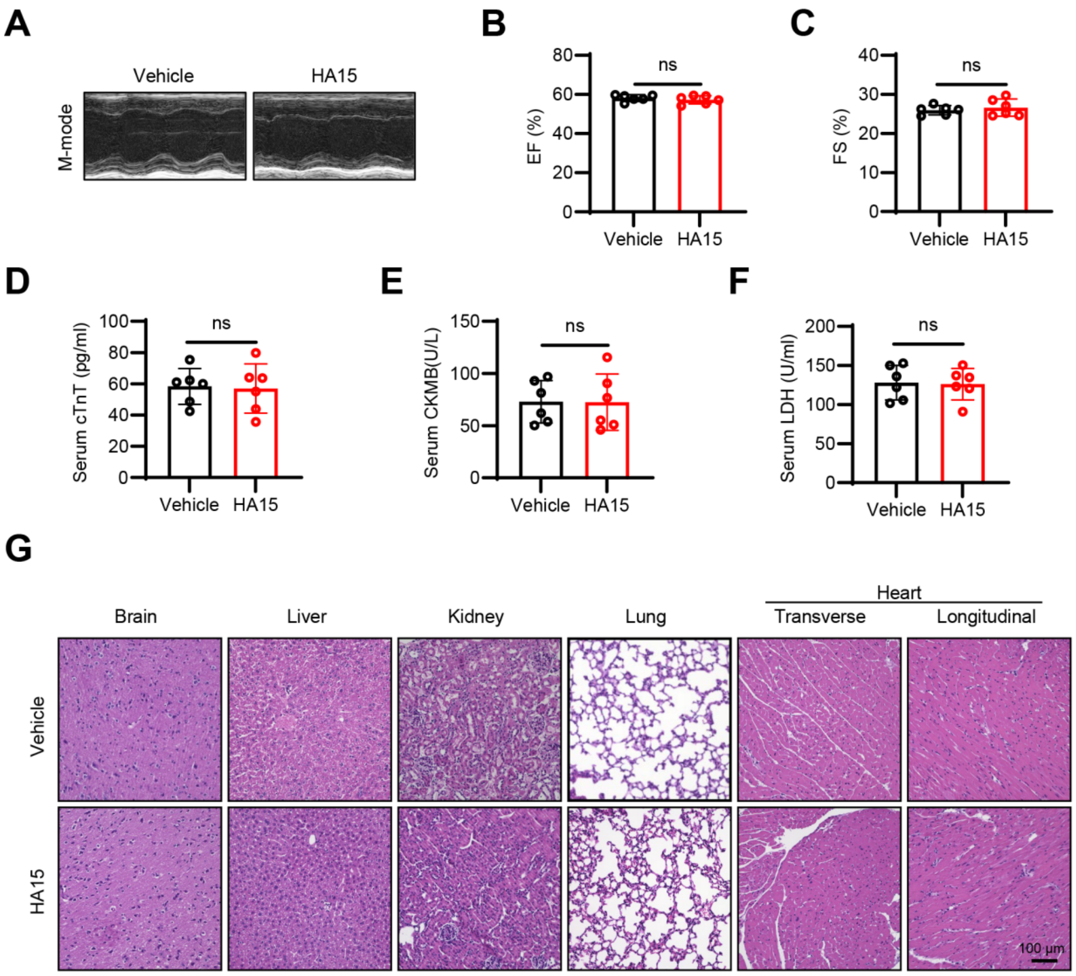

Supplementary Figure S9

(**A**) Representative M-mode echocardiography of mice in each group. Time stamp, 100 ms. Scale bar, 2mm.

(**B, C**) Myocardial function parameters, ejection fraction (EF) (**B**) and fractional shortening (FS) (**C**) of mice measured by echocardiography.

(**D-F**) Serum concentrations of creatine kinase isoenzyme MB (CK-MB) (**D**), cardiac troponin T (cTnT)(**E**), and LDH (**F**) in each group.

(**G**) H&E staining of major organs (brain, liver, kidney, lung and heart) in each group.

Data are expressed as the mean ± SD. ns, $P > 0.05$, ns: no differences. n = 6. Student's t-test for B, C, D, E and F.

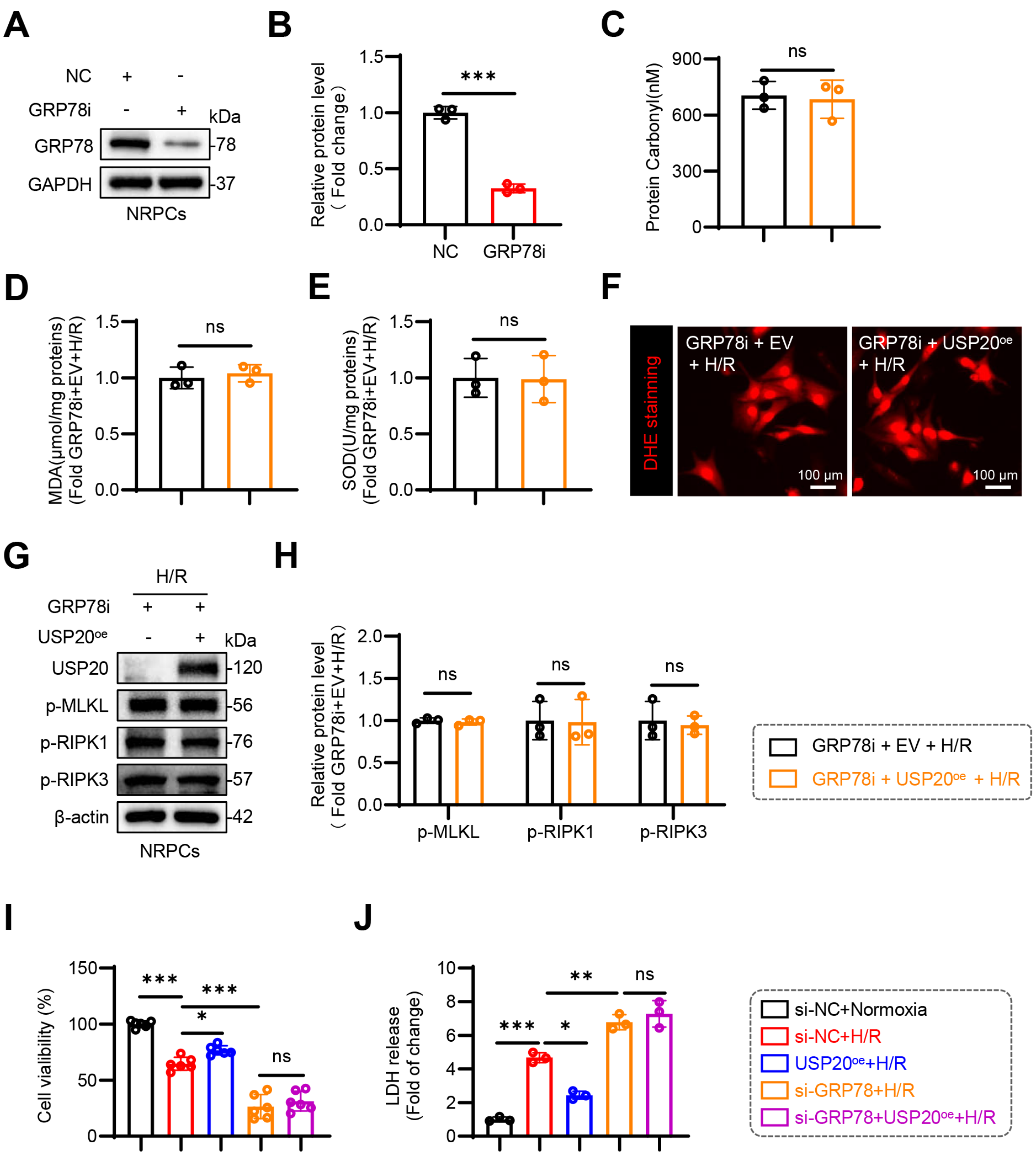

Supplementary Figure S10

(**A, B**) The protein expression level of GRP78 in NRPCs following siRNA-mediated silencing of GRP78 (**A**) and the statistical results (**B**). n = 3.

(**C-F**) The levels of protein carbonylation (**C**), MDA (**D**), SOD (**E**), and DHE (**F**) in NRPCs following GRP78 silencing and USP20 overexpression under H/R stimulation. n = 3.

(**G, H**) The protein expression levels of USP20, p-MLKL, p-RIPK1 and p-RIPK3 in NRPCs following GRP78 silencing and USP20 overexpression under H/R stimulation (**G**) and the statistical results (**H**). n = 3.

(**I, J**) CCK8 assay (**I**) and LDH release assay (**J**) showing the effects of USP20 overexpression on cell viability and cellular injury in USP20-deficient NRPCs subjected to H/R treatment.

Data are expressed as the mean ± SD. *, $P < 0.05$; **, $P < 0.01$; ***, $P < 0.001$; ns, $P > 0.05$. ns: no differences. Student's t-test for B, C, D, E and H. One-way ANOVA followed by Tukey's post hoc test for I and J.

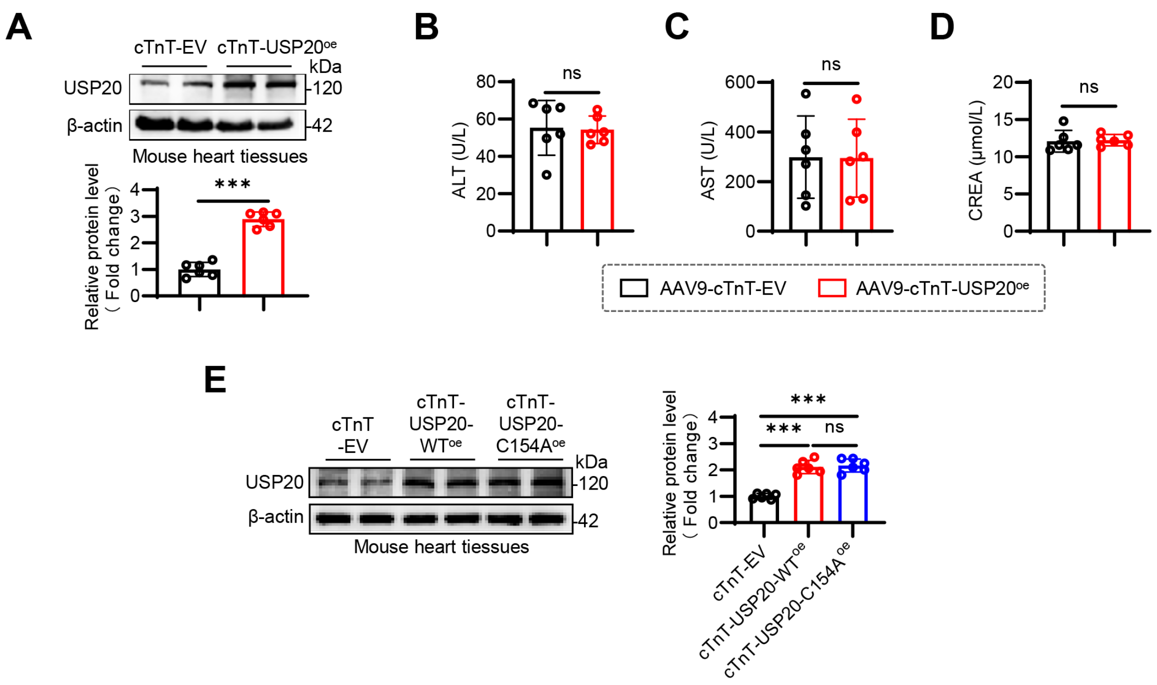

Supplementary Figure S11

(**A**) Representative western blot of USP20 in heart tissues from mice subjected to AAV9-$USP20^{WT}$ and the statistical results. n = 6.

(**B-D**) Serum concentrations of Alanine aminotransferase (ALT, **B**), Aspartate aminotransferase (AST, **C**), and Creatinine (CREA, **F**) in each group.

(**E**) Representative western blot of USP20 in heart tissues from mice subjected to AAV9-cTnT-$USP20^{WT}$ or AAV9-cTnT $USP20^{C154A}$ and the statistical results. n = 6.

Data are expressed as the mean ± SD. ***, $P < 0.001$; ns, $P > 0.05$. ns: no differences. Student's t-test for A, B, C and D.

Supplementary Tables

Table S1: Sequences of primers for real-time qPCR assay used in the study

Gene	Species	Sequence (Forward)	Sequence (Reverse)
Usp20	Mouse	GCCTTCATCGTGGAGTACATCAGAC	CAGCAGCAAAGAAAGCAGCAAGG
Col-1	Mouse	TGGCCTTGGAGGAAACTTTG	CACAGCTTCTCTTTGATGTCAC
Tgf-β	Mouse	CCAGATCCTGTCCAAACTAAGG	CACAGCTTCTCTTTGATGTCAC
Grp78	Mouse	CCGAGGAGGAGGACAAGAAGGAG	GAACACACCGACGCAGGAATAGG
Zup1	Mouse	CCTGGATTGGAGCGTGTGAGAT	GGAGTTCCTTCCGTCTCTGAAG
Vcpip1	Mouse	CGTGTGGTCTTATGCAAAGGGAC	TGCCAGGTAGATGTGGAAAAGTAC
Uchl3	Mouse	CAAACCATCAGCAATGCCTGTGG	GGGCTCATTGATACAGACTCCTC
Usp32	Mouse	GGAAATGCCTGTATTGCTGTGGATTG	CTTGTGCTCGCCGACTCTGTTC
Usp1	Mouse	AGTAGCGTCACACCTGTGGACA	GCTTTCACATTCCAAACACCGAG
Tnfaip3	Mouse	CATAGAGACATGCCTCGAACTA	CTTGTGCTCGCCGACTCTGTTC
Usp27x	Mouse	ACCAAGGAACCTTGGAGAGTGG	CCTTCACTGTCCAGCACATCCT
Mindy3	Mouse	CTCGGTATCCATGAACAAGCAGC	GTGAGTTTCGCTGCCAACAATCC
Otud5	Mouse	TACAACCGTCCTGTGGAGGTGT	TGGTAGCTGACACGGATGGGTT
Usp35	Mouse	AAGGAGCTGTTCGCTGTCATC	TCATCCTGCTAATGGCAGTCA
Usp21	Mouse	TCCTGAATGCCGTGCTACAGTG	AGAGGGCACCAATCACATCTGC
Otud7a	Mouse	CTCATCCTGTCCCTAGAAGCCA	GTTGGAGACTCTGGCTGTGCAA
Usp2	Mouse	ACAACCTGTATGCTGTGTCCAATC	GTGTGCCATTCGCCTGTAACC
Usp36	Mouse	CGATGTGGTCCAGGCGTTGC	AAGGCTCGCCATCCCAGGTC
Usp54	Mouse	ATCCGCCGTGTCTTGATGAATG	GTCCTCTGCCAAGTCTGAATGC
β-actin	Mouse	CCGTGAAAAGATGACCCAGA	TACGACCAGAGGCATACAG
Usp20	Rat	GCAAGTGGATGAGGACGCTGATG	GTGGACTTCATTGTCTGGCTCTGG
β-actin	Rat	CCTAGACTTCGAGCAAGAGA	GGAAGGAAGGCTGGAAGA
Usp20	Human	TTCTACAGGAAGAGCAGCGAGGAG	GCGAAGGTGTTGAACTTGTTGAGC
β-actin	Human	CCTGGCACCCAGCACAAT	GCCGATCCACACGGAGTACT

Table S2: Biometric and echocardiographic parameters in mice of each group

**Chronic myocardial I/R injury model**	**Sham Group**		**I/R Group**	
**Parameter**	**USP20$^{fl/fl}$ n=6**	**USP20 CKO n=6**	**USP20$^{fl/fl}$ n=6**	**USP20 CKO n=6**
Heart rate, (bpm)	507±20	499±28^{ns}	527±20^{ns}	502±24^{NS}
LVAW; s, (mm)	1.17±0.21	1.18±0.14^{ns}	1.15±0.12^{ns}	0.87±0.16$^{\#}$
LVAW; d, (mm)	0.56±0.14	0.62±0.15^{ns}	0.81±0.11*	0.69±0.10^{NS}
LVPW; s, (mm)	1.25±0.25	1.11±0.28^{ns}	0.96±0.23^{ns}	1.11±0.22^{NS}
LVPW; d, (mm)	0.89±0.28	0.66±0.26^{ns}	0.74±0.25^{ns}	0.98±0.30^{NS}

ns and *, represents USP20 CKO + Sham group or USP20$^{fl/fl}$ + I/R group compared with USP20$^{fl/fl}$ + Sham group. *, $P < 0.05$; ns, $P > 0.05$. ns, no significance.

NS and #, represents USP20 CKO + I/R group compared with USP20$^{fl/fl}$ + I/R group. #, $P < 0.05$; NS, $P > 0.05$. NS, no significance.
